# Supplementary material for: Antioxidant, Cytotoxic, and Rheological Properties of Canola Oil Extract of Usnea barbata (L.) Weber ex F.H. Wigg from Călimani Mountains, Romania
Source: Plants (Basel). 2022 Mar 23;11(7):854. doi: 10.3390/plants11070854 (PMC9002375; doi:10.3390/plants11070854)
Supplement: Supplementary file 1 [file plants-11-00854-s001.zip › UHPLC Specificity.pdf]

Sample Replicate Report - Multi-Channel

Sample Name	UA std 50 ug/mL
Batch Group/Name	UMF Ovidius/20211109 Usnea barbata oil extract - Copy 11-10-2021 08-56-41 - Copy 11-10-2021 09-15-52 - (

Acquisition Method	20211103 Usnic Acid Oil		
Processing Method	20211103 Usnic Acid Oil		
Instrument Name	HPLC-PDA Plus		
Vial Number	8	Operator	dan.rambu
Acquisition Date	11/9/2021	Chromera Version	4.2.0.6415

UA std 50 ug/mL : 320:10:400:10 : 1

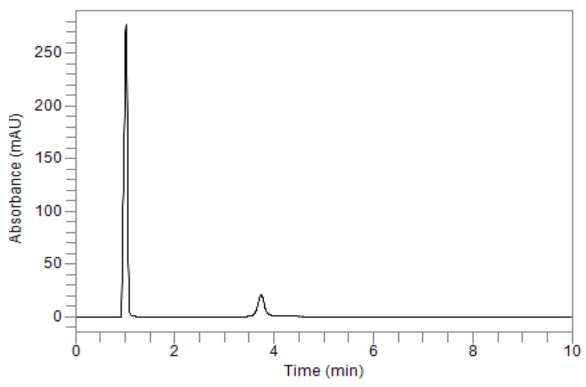

UA std 50 ug/mL : 320:10:400:10 : 2

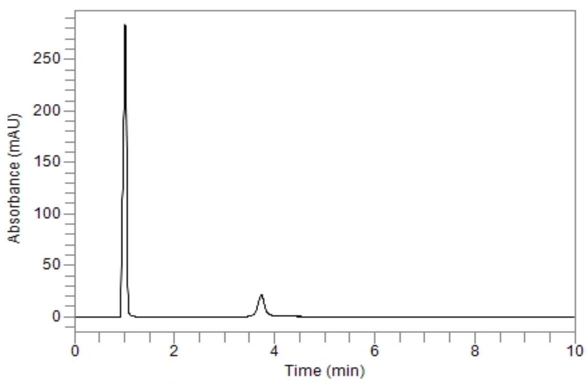

UA std 50 ug/mL : 320:10:400:10 : 3

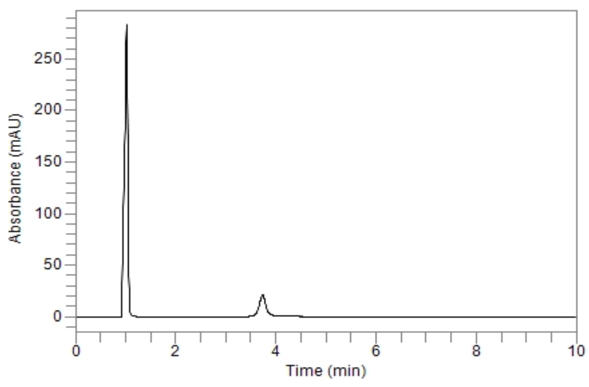

UA std 50 ug/mL : 320:10:400:10 : 4

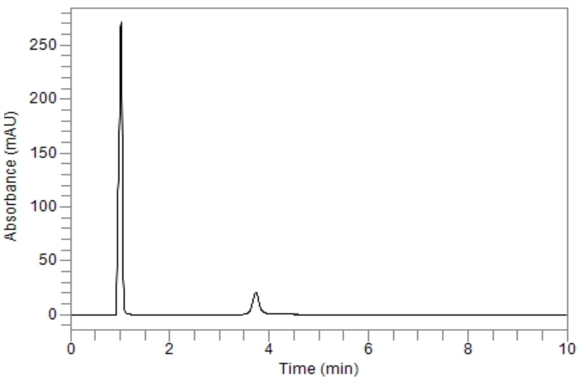

UA std 50 ug/mL : 320:10:400:10 : 5

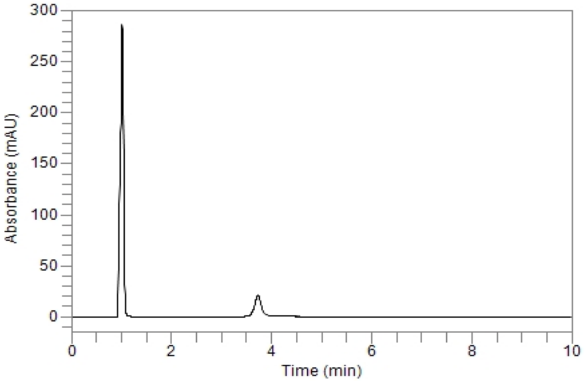

Channel Name **320:10:400:10**

Component Name	Avg RT (min)	RT %RSD	Avg Area	Area %RSD	Avg Height	Height %RSD	Avg Final Amount	Final %RSD
	N/A	N/A	N/A	N/A	N/A	N/A	N/A	N/A

UA std 50 ug/mL : 282:10:400:10 : 1

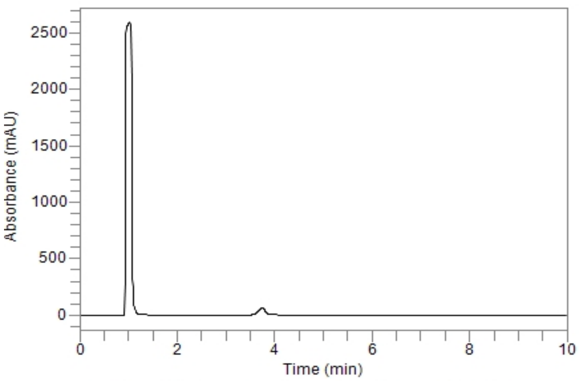

UA std 50 ug/mL : 282:10:400:10 : 2

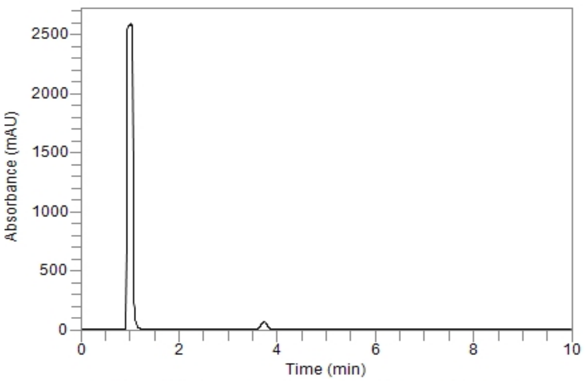

UA std 50 ug/mL : 282:10:400:10 : 3

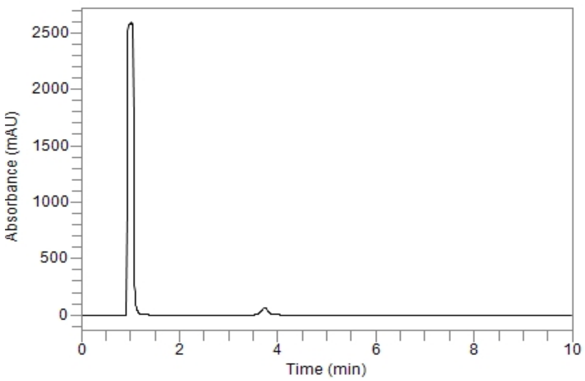

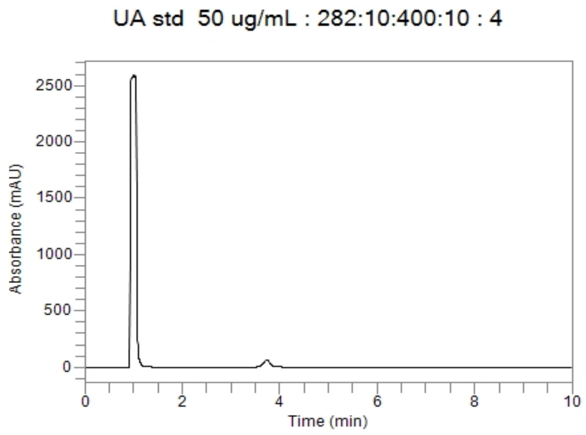

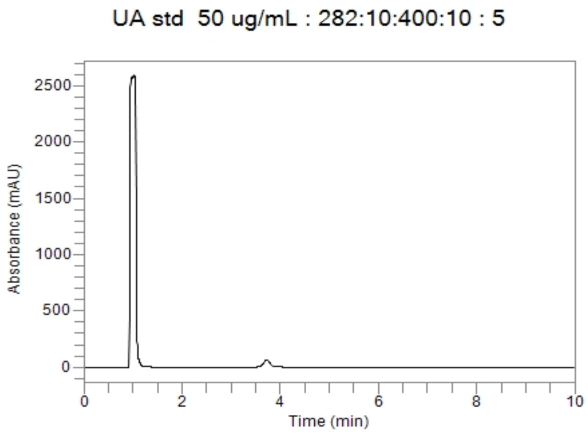

Channel Name **282:10:400:10**

Component Name	Avg RT (min)	RT %RSD	Avg Area	Area %RSD	Avg Height	Height %RSD	Avg Final Amount	Final %RSD
Usnic Acid	3.735	0.115	712,154.0	2.152	67,653.2	0.845	49.8148	2.115

Copy 11-10-

Sample Replicate Report - Multi-Channel

Sample Name	UBO 10 mg/ML		
Batch Group/Name	UMF Ovidius/20211109 Usnea barbata oil extract - Copy 11-10-2021 08-56-41 - Copy 11-10-2021 09-15-52 - (		
Acquisition Method	20211103 Usnic Acid Oil		
Processing Method	20211103 Usnic Acid Oil		
Instrument Name	HPLC-PDA Plus		
Vial Number	10	Operator	dan.rambu
Acquisition Date	11/9/2021	Chromera Version	4.2.0.6415

UBO 10 mg/ML : 320:10:400:10 : 1

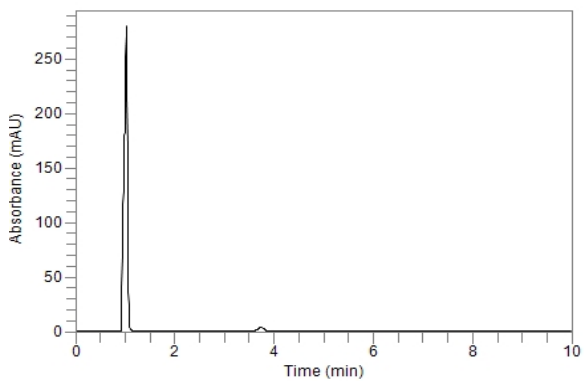

Channel Name **320:10:400:10**

Component Name	Avg RT (min)	RT %RSD	Avg Area	Area %RSD	Avg Height	Height %RSD	Avg Final Amount	Final %RSD
	N/A	N/A	N/A	N/A	N/A	N/A	N/A	N/A

UBO 10 mg/ML : 282:10:400:10 : 1

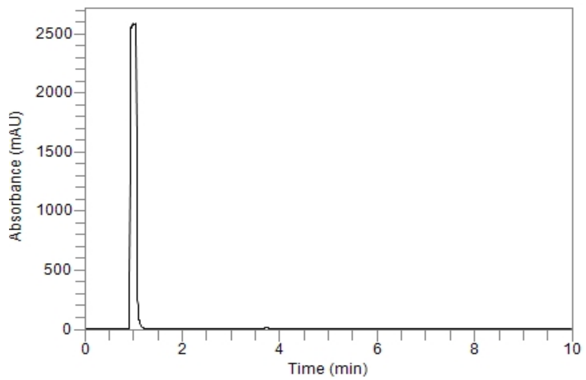

Channel Name **282:10:400:10**

Component Name	Avg RT (min)	RT %RSD	Avg Area	Area %RSD	Avg Height	Height %RSD	Avg Final Amount	Final %RSD
Usnic Acid	3.735	N/A	130,381.4	N/A	12,729.3	N/A	9.8288	N/A

Copy 11-10-

Sample Replicate Report - Multi-Channel

Sample Name	Canola Oil		
Batch Group/Name	UMF Ovidius/20211109 Usnea barbata oil extract - Copy 11-10-2021 08-56-41 - Copy 11-10-2021 09-15-52 - (		
Acquisition Method	20211103 Usnic Acid Oil		
Processing Method	20211103 Usnic Acid Oil		
Instrument Name	HPLC-PDA Plus		
Vial Number	2	Operator	dan.rambu
Acquisition Date	11/9/2021	Chromera Version	4.2.0.6415

Canola Oil : 320:10:400:10 : 1

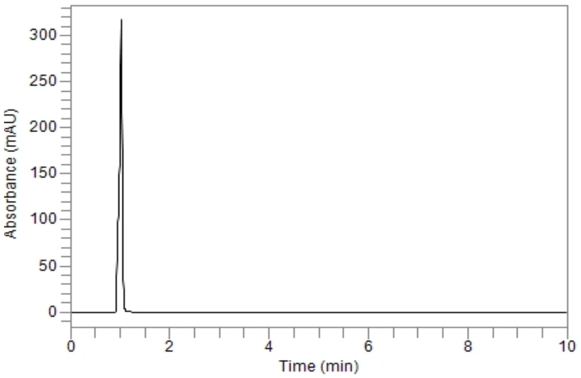

Channel Name **320:10:400:10**

Component Name	Avg RT (min)	RT %RSD	Avg Area	Area %RSD	Avg Height	Height %RSD	Avg Final Amount	Final %RSD
	N/A	N/A	N/A	N/A	N/A	N/A	N/A	N/A

Canola Oil : 282:10:400:10 : 1

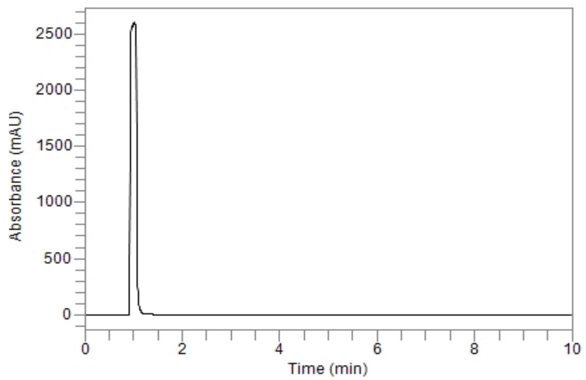

Channel Name **282:10:400:10**

Component Name	Avg RT (min)	RT %RSD	Avg Area	Area %RSD	Avg Height	Height %RSD	Avg Final Amount	Final %RSD
	N/A	N/A	N/A	N/A	N/A	N/A	N/A	N/A

:opy 11-10-

Sample Report - Single Channel

Sample Name	Acetone		
Batch Group/Name	UMF Ovidius/20211109 Usnea barbata oil extract - Copy 11-10-2021 08-56-4		
Acquisition Method	20211103 Usnic Acid Oil		
Processing Method	20211103 Usnic Acid Oil		
Instrument Name	HPLC-PDA Plus	Channel Name	320:10:400:10
Vial Number	1	Injection Number	2
Operator	dan.rambu	Chromera Version	4.2.0.6415
Acquisition Date/Time	11.9.2021 4:17:34 PM		

Acetone : Injection 2

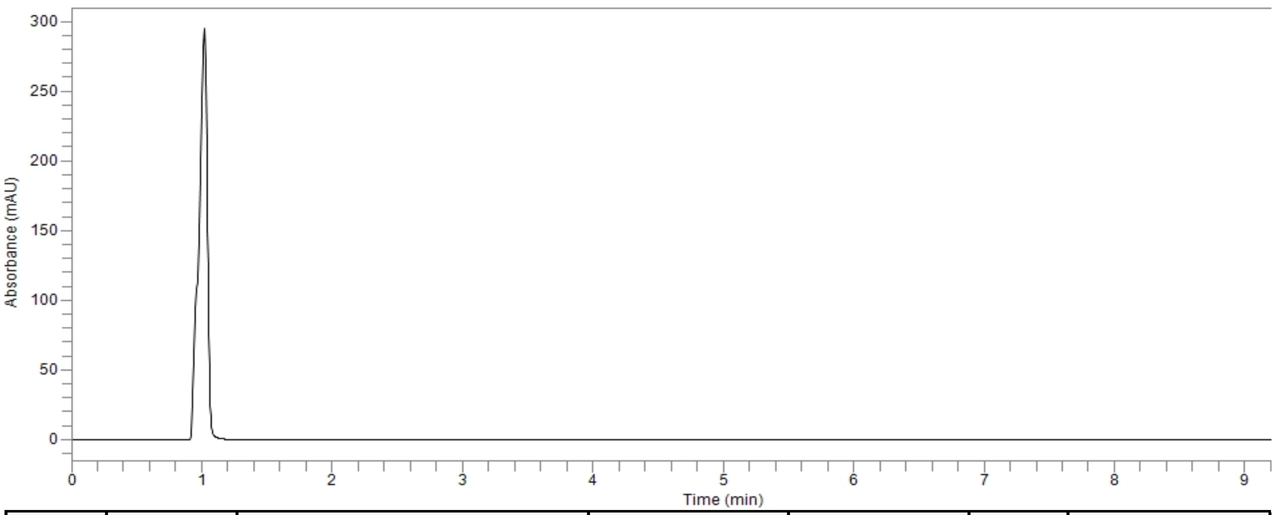

Peak #	RT (min)	Component Name	Area	Height	BL	Final Amount
1	0.545		332.0	98.8	BB	
2	1.017		1,365,047.6	295,017.5	BB	
**Total**			1,365,379.6			

1 - Copy 11-

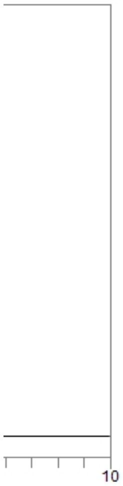

Units

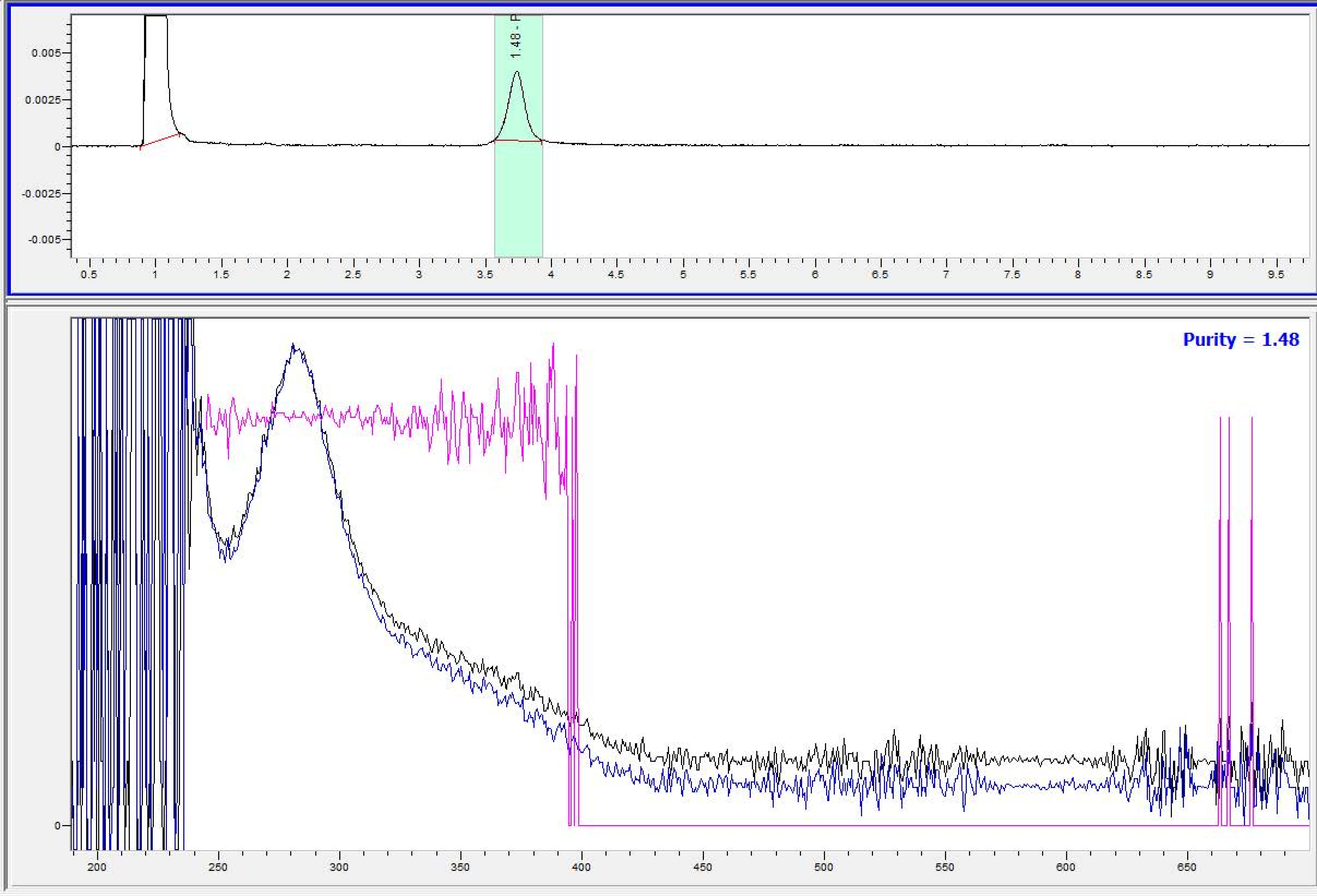
1.48 - F
0.005
0.0025
0
-0.0025
-0.005
0.5
1
1.5
2
2.5
3
3.5
4
4.5
5
5.5
6
6.5
7
7.5
8
8.5
9
9.5
Purity = 1.48
200
250
300
350
400
450
500
550
600
650

Display
☑ Upslope
☑ Downslope
☐ Baseline
☑ Purity Result
