# Supplementary material for: Antioxidant, Cytotoxic, and Rheological Properties of Canola Oil Extract of Usnea barbata (L.) Weber ex F.H. Wigg from Călimani Mountains, Romania
Source: Plants (Basel). 2022 Mar 23;11(7):854. doi: 10.3390/plants11070854 (PMC9002375; doi:10.3390/plants11070854)
Supplement: Supplementary file 1 [file plants-11-00854-s001.zip › UHPLC Accuracy and recovery.pdf]

# Sample Report - Multi-Channel

| | |
|---|---|
| Sample Name | QC 7.5 ug/mL |
| Batch Group/Name | UMF Ovidius/20211109 Usnea barbata oil extract - Copy 11-10-202 |

| | | | |
|---|---|---|---|
| Acquisition Date/Time | 11.9.2021 10:19:54 PM | | |
| Acquisition Method | 20211103 Usnic Acid Oil | | |
| Processing Method | 20211103 Usnic Acid Oil | | |
| Instrument Name | HPLC-PDA Plus | | |
| Vial Number | 9 | Injection Number | 1 |
| Operator | dan.rambu | Chromera Version | 4.2.0.6 |

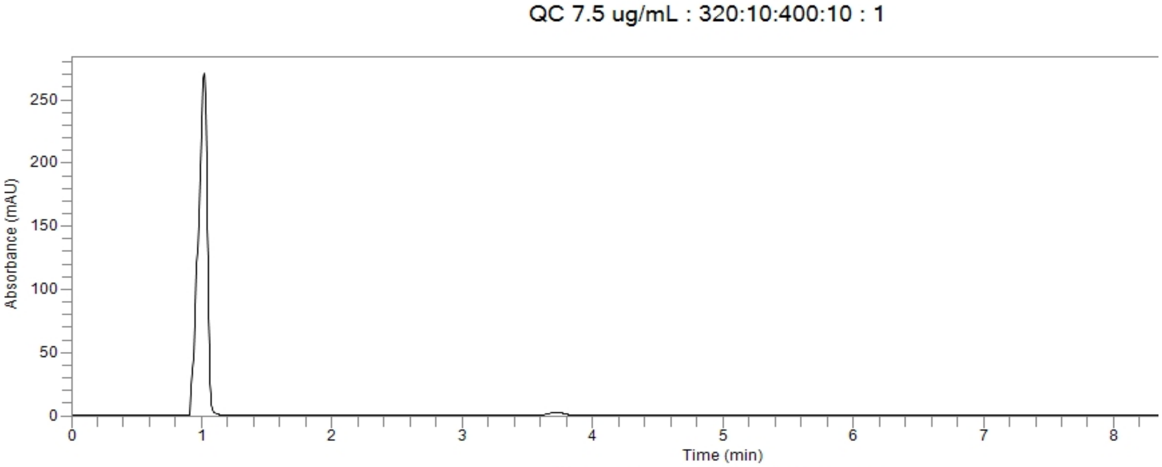


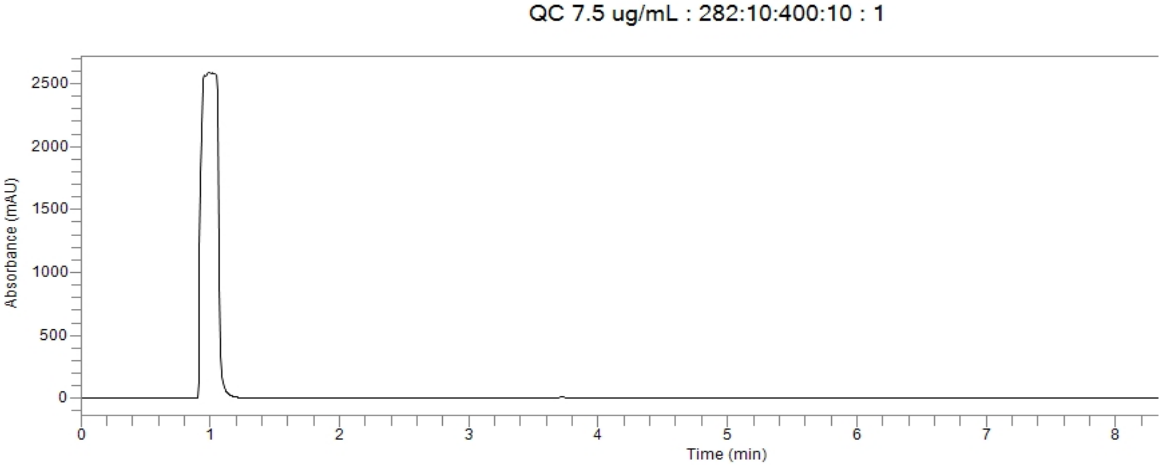


| Peak # | RT (min) | Component Name | Channel | Area | Height | BL |
|---|---|---|---|---|---|---|
| 1 | 0.997 | | 282:10:400:10 | 22,677,961 | 2,590,940 | BB |

| | | | | | | |
|---|---|---|---|---|---|---|
| 1 | 1.016 | | 320:10:400:10 | 1,344,208 | 271,096 | BB |
| 2 | 3.723 | Usnic Acid | 282:10:400:10 | 88,436 | 8,764 | BB |
| 2 | 3.724 | | 320:10:400:10 | 24,739 | 2,682 | BB |
| **Total** | | | | 24,135,343 | | |

1 08-56-41 - Copy 11-

415

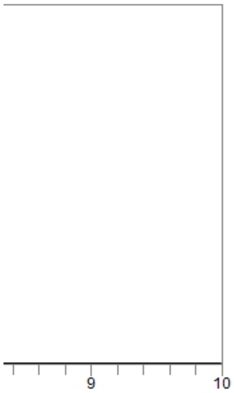


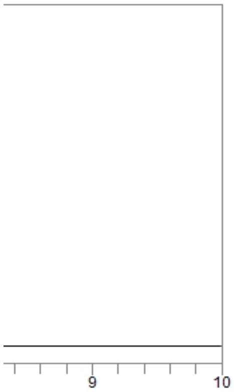


| Final Amount | Units |
|---|---|
| | |

| | |
|---|---|
| | |
| 6,9458 | µg/mL |
| | |
| 6.9458 | |

# Sample Report - Multi-Channel

| | | | |
|---|---|---|---|
| Sample Name | QC 7.5 ug/mL | | |
| Batch Group/Name | UMF Ovidius/20211109 Usnea barbata oil extract - Copy 11-10-202 | | |
| Acquisition Date/Time | 11.9.2021 10:54:58 PM | | |
| Acquisition Method | 20211103 Usnic Acid Oil | | |
| Processing Method | 20211103 Usnic Acid Oil | | |
| Instrument Name | HPLC-PDA Plus | | |
| Vial Number | 9 | Injection Number | 4 |
| Operator | dan.rambu | Chromera Version | 4.2.0.6 |

QC 7.5 ug/mL : 320:10:400:10 : 4

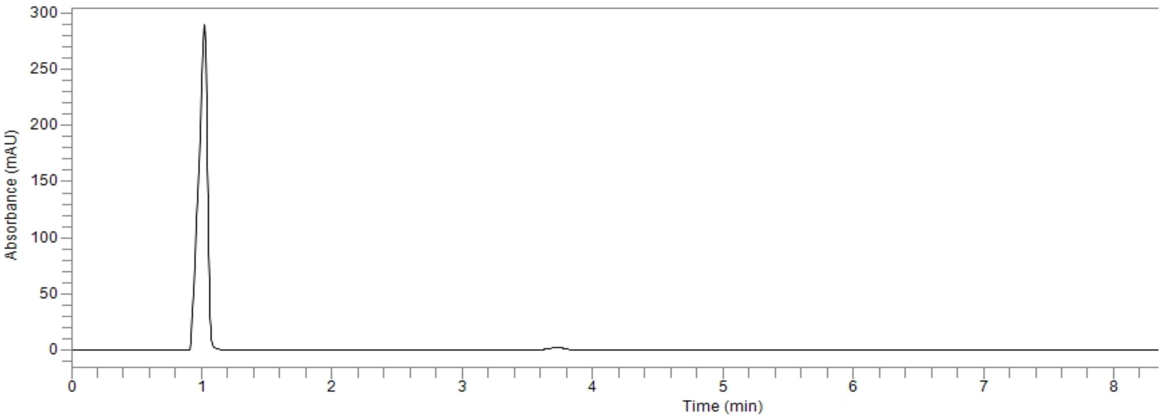


QC 7.5 ug/mL : 282:10:400:10 : 4

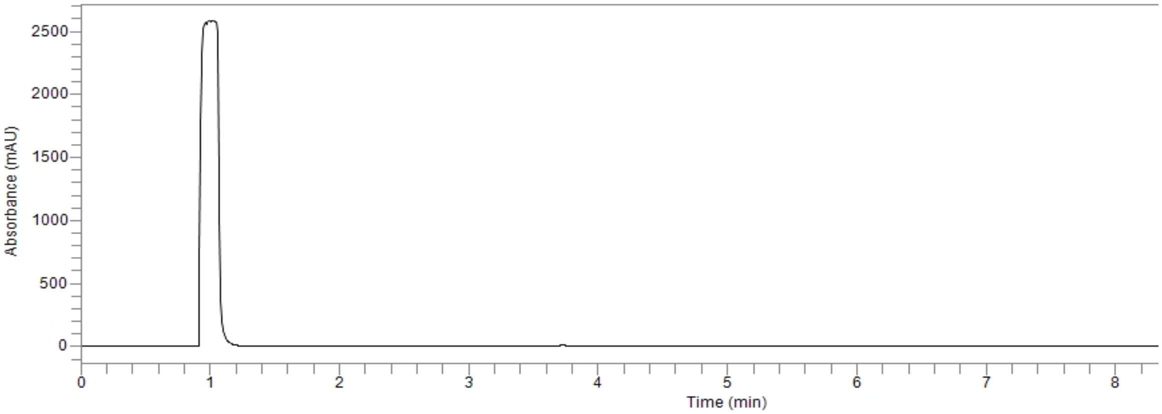


| Peak # | RT (min) | Component Name | Channel | Area | Height | BL |
|---|---|---|---|---|---|---|
| 1 | 0.995 | | 282:10:400:10 | 22,603,492 | 2,585,938 | BB |

| | | | | | | |
|---|---|---|---|---|---|---|
| 1 | 1.019 | | 320:10:400:10 | 1,405,142 | 290,302 | BB |
| 2 | 3.727 | | 320:10:400:10 | 27,012 | 2,765 | BB |
| 2 | 3.728 | Usnic Acid | 282:10:400:10 | 92,735 | 8,982 | BB |
| **Total** | | | | 24,128,381 | | |

1 08-56-41 - Copy 11-

415

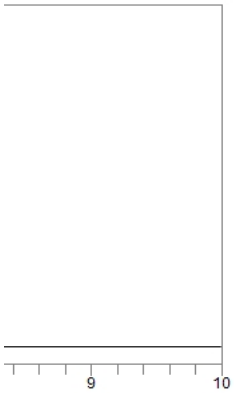


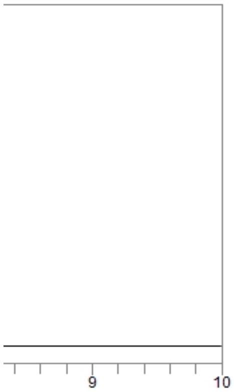


| Final Amount | Units |
|---|---|
| | |

| | |
|---|---|
| | |
| | |
| 7,2413 | µg/mL |
| 7.2413 | |

# Sample Report - Multi-Channel

| | | | |
|---|---|---|---|
| Sample Name | QC 7.5 ug/mL | | |
| Batch Group/Name | UMF Ovidius/20211109 Usnea barbata oil extract - Copy 11-10-202 | | |
| Acquisition Date/Time | 11.9.2021 11:06:39 PM | | |
| Acquisition Method | 20211103 Usnic Acid Oil | | |
| Processing Method | 20211103 Usnic Acid Oil | | |
| Instrument Name | HPLC-PDA Plus | | |
| Vial Number | 9 | Injection Number | 5 |
| Operator | dan.rambu | Chromera Version | 4.2.0.6 |

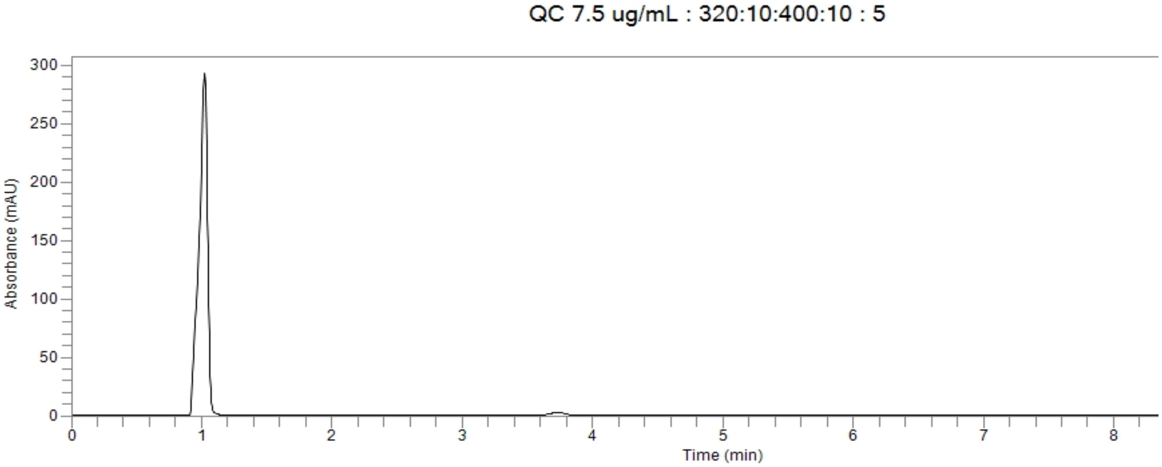


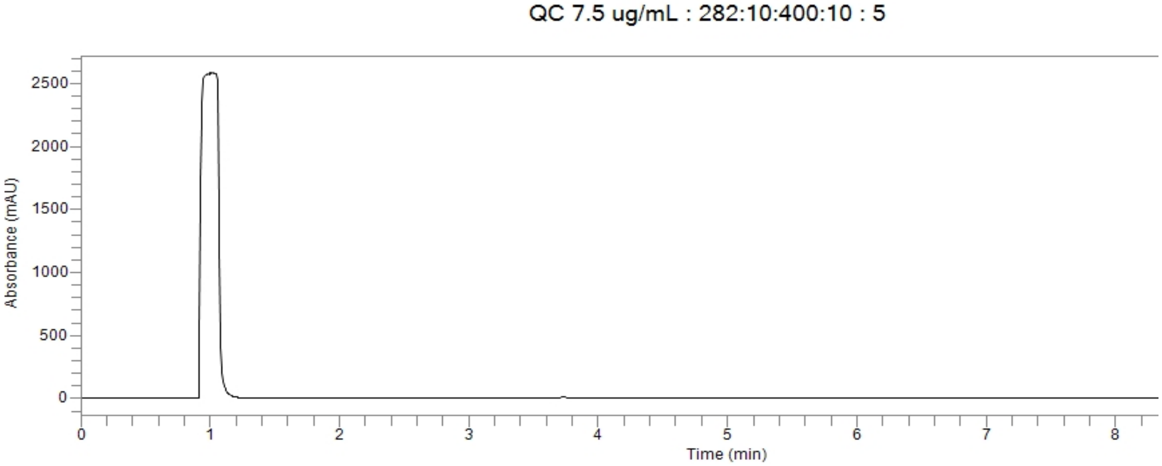


| Peak # | RT (min) | Component Name | Channel | Area | Height | BL |
|---|---|---|---|---|---|---|
| 1 | 1.020 | | 320:10:400:10 | 1,376,726 | 293,043 | BB |

| | | | | | | |
|---|---|---|---|---|---|---|
| 1 | 1.021 | | 282:10:400:10 | 22,656,659 | 2,589,605 | BB |
| 2 | 3.736 | Usnic Acid | 282:10:400:10 | 91,150 | 8,897 | BB |
| 2 | 3.740 | | 320:10:400:10 | 25,681 | 2,710 | BB |
| **Total** | | | | 24,150,217 | | |

1 08-56-41 - Copy 11-

415

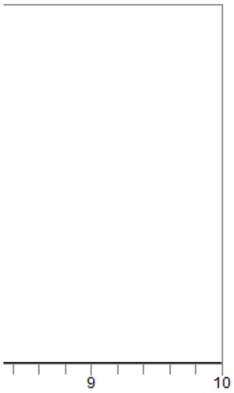


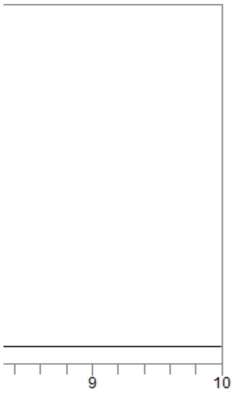


| Final Amount | Units |
| --- | --- |
| | |

| | |
|---|---|
| | |
| 7,1324 | µg/mL |
| | |
| 7.1324 | |

# Sample Report - Multi-Channel

| | | | |
|---|---|---|---|
| Sample Name | QC 7.5 ug/mL | | |
| Batch Group/Name | UMF Ovidius/20211109 Usnea barbata oil extract - Copy 11-10-202 | | |
| Acquisition Date/Time | 11.9.2021 11:18:20 PM | | |
| Acquisition Method | 20211103 Usnic Acid Oil | | |
| Processing Method | 20211103 Usnic Acid Oil | | |
| Instrument Name | HPLC-PDA Plus | | |
| Vial Number | 9 | Injection Number | 6 |
| Operator | dan.rambu | Chromera Version | 4.2.0.6 |

QC 7.5 ug/mL : 320:10:400:10 : 6

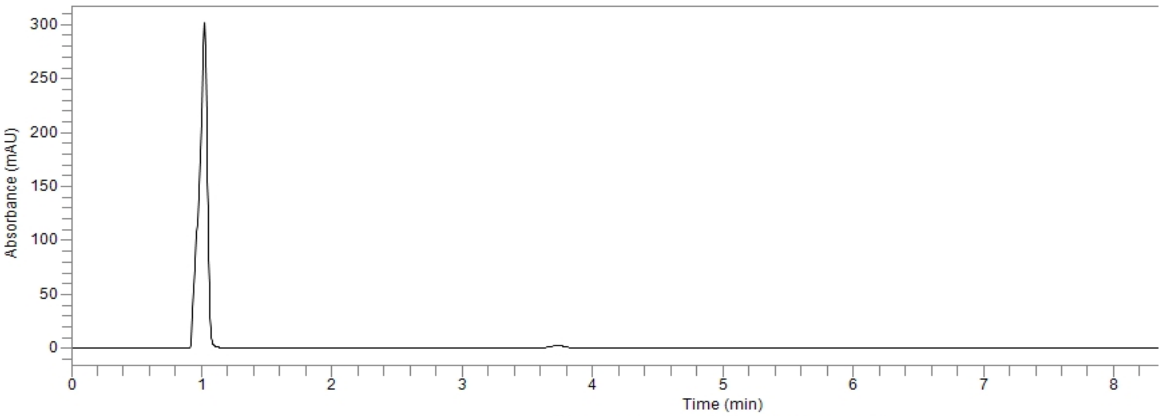


QC 7.5 ug/mL : 282:10:400:10 : 6

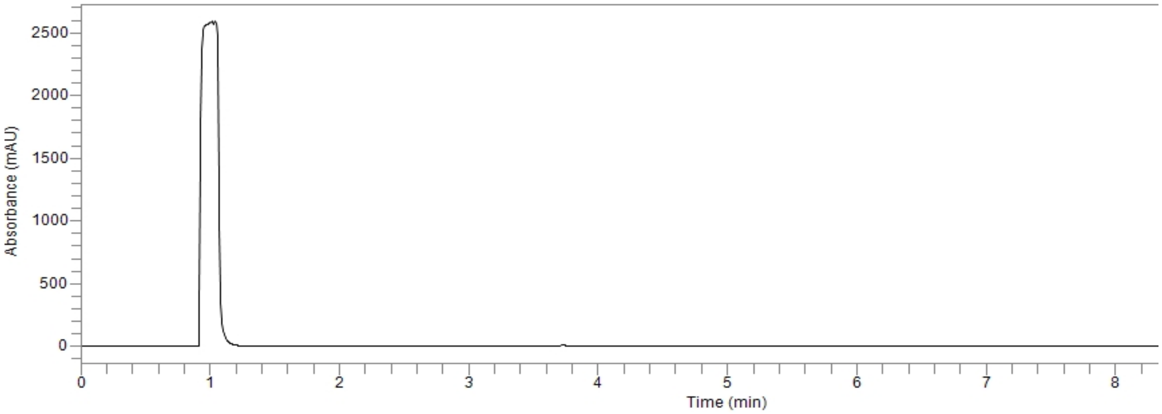


| Peak # | RT (min) | Component Name | Channel | Area | Height | BL |
|---|---|---|---|---|---|---|
| 1 | 1.013 | | 282:10:400:10 | 22,336,008 | 2,594,293 | BB |

| | | | | | | |
|---|---|---|---|---|---|---|
| 1 | 1.018 | | 320:10:400:10 | 1,349,530 | 302,955 | BB |
| 2 | 3.732 | Usnic Acid | 282:10:400:10 | 89,034 | 8,775 | BB |
| 2 | 3.734 | | 320:10:400:10 | 24,395 | 2,653 | BB |
| **Total** | | | | 23,798,967 | | |

1 08-56-41 - Copy 11-

415

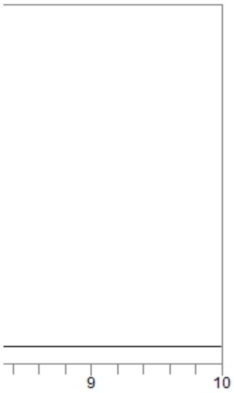


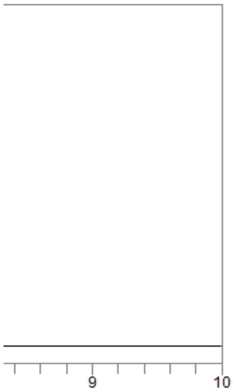


| Final Amount | Units |
| --- | --- |
| | |

| | |
|---|---|
| 6,9869 | µg/mL |
| | |
| 6.9869 | |

# Sample Report - Multi-Channel

| | | | |
|---|---|---|---|
| Sample Name | QC 7.5 ug/mL | | |
| Batch Group/Name | UMF Ovidius/20211109 Usnea barbata oil extract - Copy 11-10-202 | | |
| Acquisition Date/Time | 11.9.2021 10:31:36 PM | | |
| Acquisition Method | 20211103 Usnic Acid Oil | | |
| Processing Method | 20211103 Usnic Acid Oil | | |
| Instrument Name | HPLC-PDA Plus | | |
| Vial Number | 9 | Injection Number | 2 |
| Operator | dan.rambu | Chromera Version | 4.2.0.6 |

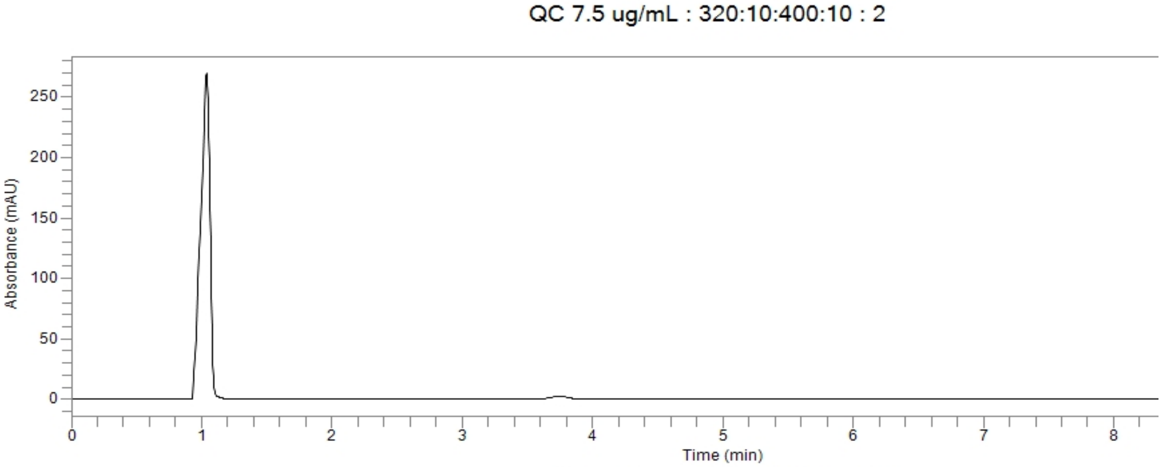


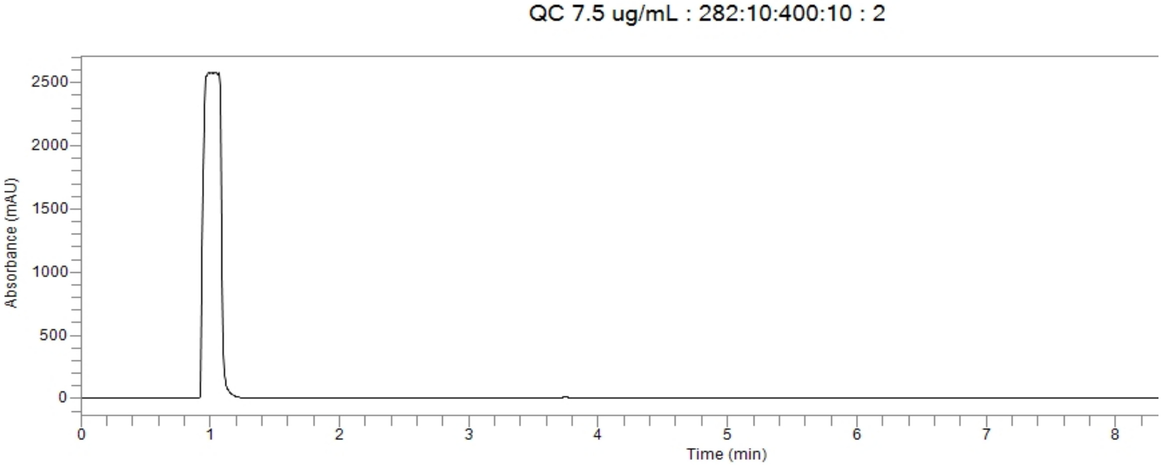


| Peak # | RT (min) | Component Name | Channel | Area | Height | BL |
|---|---|---|---|---|---|---|
| 1 | 1.037 | | 320:10:400:10 | 1,398,859 | 270,477 | BB |

| | | | | | | |
|---|---|---|---|---|---|---|
| 1 | 1.042 | | 282:10:400:10 | 22,904,320 | 2,584,995 | BB |
| 2 | 3.749 | Usnic Acid | 282:10:400:10 | 90,957 | 8,901 | BB |
| 2 | 3.750 | | 320:10:400:10 | 25,569 | 2,735 | BB |
| **Total** | | | | 24,419,705 | | |

1 08-56-41 - Copy 11-

415

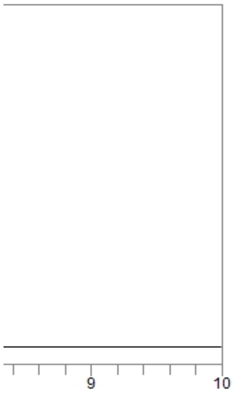


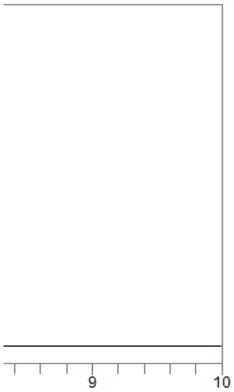


| Final Amount | Units |
|---|---|
| | |

| | |
|---|---|
| | |
| 7,1191 | µg/mL |
| | |
| 7.1191 | |

# Sample Report - Multi-Channel

| | | | |
|---|---|---|---|
| Sample Name | QC 7.5 ug/mL | | |
| Batch Group/Name | UMF Ovidius/20211109 Usnea barbata oil extract - Copy 11-10-202 | | |
| Acquisition Date/Time | 11.9.2021 10:43:17 PM | | |
| Acquisition Method | 20211103 Usnic Acid Oil | | |
| Processing Method | 20211103 Usnic Acid Oil | | |
| Instrument Name | HPLC-PDA Plus | | |
| Vial Number | 9 | Injection Number | 3 |
| Operator | dan.rambu | Chromera Version | 4.2.0.6 |

QC 7.5 ug/mL : 320:10:400:10 : 3

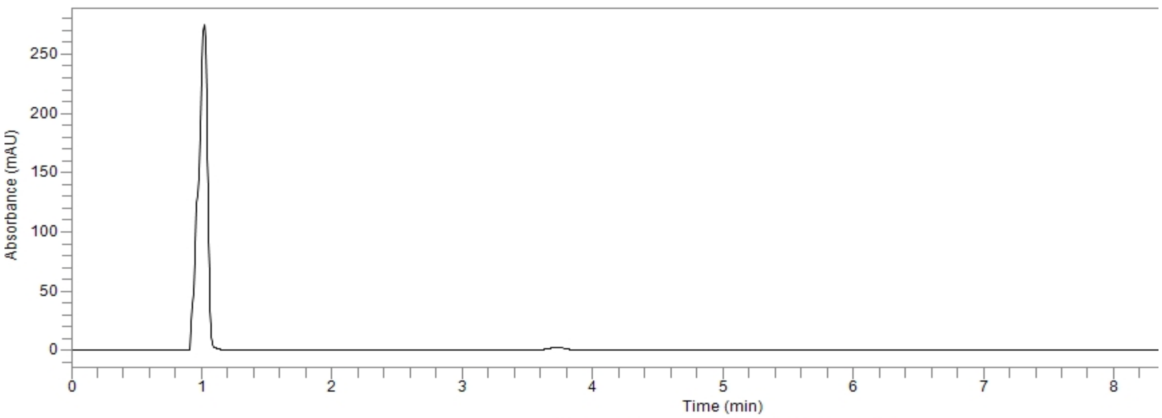


QC 7.5 ug/mL : 282:10:400:10 : 3

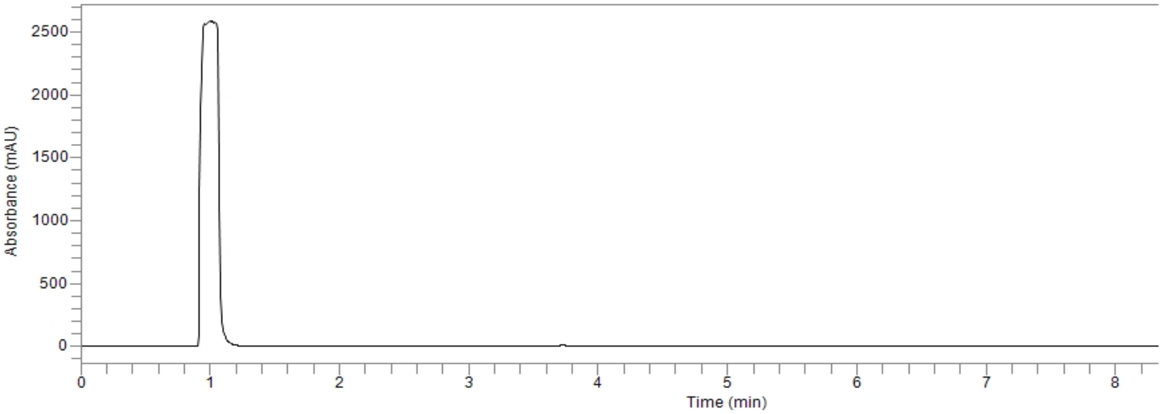


| Peak # | RT (min) | Component Name | Channel | Area | Height | BL |
|---|---|---|---|---|---|---|
| 1 | 1.014 | | 282:10:400:10 | 23,080,954 | 2,592,497 | BB |

| | | | | | | |
|---|---|---|---|---|---|---|
| 1 | 1.019 | | 320:10:400:10 | 1,388,632 | 274,288 | BB |
| 2 | 3.729 | Usnic Acid | 282:10:400:10 | 91,862 | 8,883 | BB |
| 2 | 3.731 | | 320:10:400:10 | 25,149 | 2,701 | BB |
| **Total** | | | | 24,586,598 | | |

1 08-56-41 - Copy 11-

415

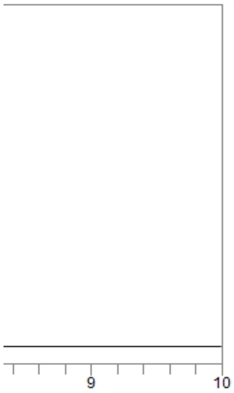


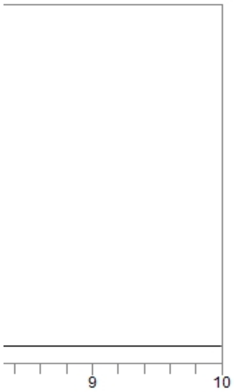


| Final Amount | Units |
|---|---|
| | |

| | |
|---|---|
| | |
| 7.1813 | µg/mL |
| | |
| 7,1813 | |

UA 10ug rec : 282:10:400:10 : 1

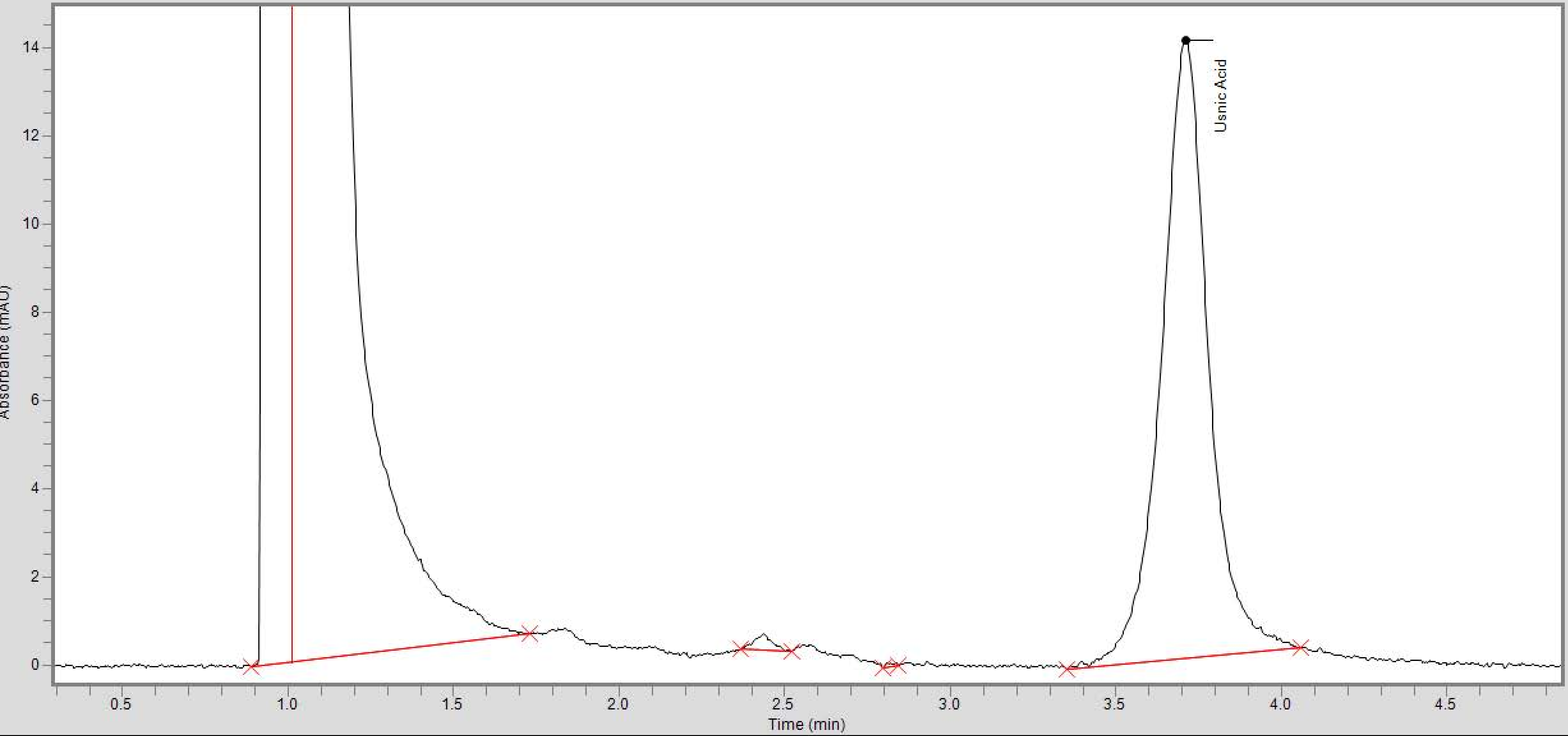


**Review Calibration** *Apply Calibration* **Review Samples** **Set 1 of 1**

| Channel | Ret. Time | Component Name | Area | Height | Final Amount | Final Amount Units | Peak Purity | Peak Purity Details |
|---|---|---|---|---|---|---|---|---|
| 320:10:400:10 | 3.711 | | 33108.92 | 3996.22 | | | 9.71 | Fail |
| 320:10:400:10 | 4.639 | | 70.02 | 79.31 | | | 4.19 | Fail |
| 320:10:400:10 | 9.370 | | 184.77 | 129.88 | | | 9.89 | Fail |
| 282:10:400:10 | 0.984 | | 12993000.56 | 2588509.11 | | | | |
| 282:10:400:10 | 1.036 | | 10069460.86 | 2606439.88 | | | | |
| 282:10:400:10 | 2.437 | | 1399.10 | 364.72 | | | | |
| 282:10:400:10 | 2.833 | | 161.59 | 68.28 | | | | |
| 282:10:400:10 | 3.394 | | 245.92 | 145.89 | | | | |
| 282:10:400:10 | 3.711 | Usnic Acid | 138527.18 | 14029.27 | 10.388648 | µg/mL | | |
| 282:10:400:10 | 6.487 | | 383.77 | 144.12 | | | | |

UA 10ug rec : 282:10:400:10 : 2

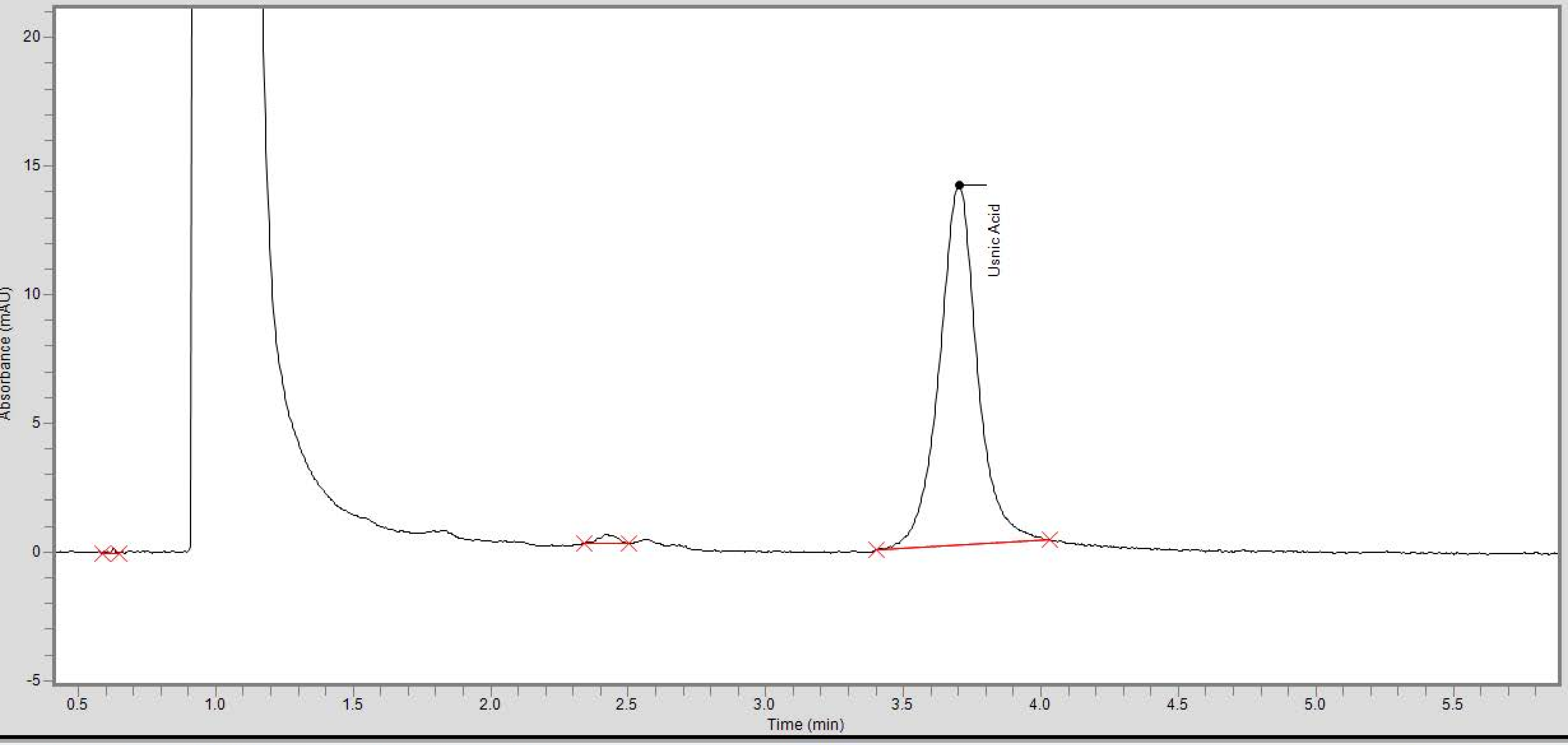


**Review Calibration** *Apply Calibration* **Review Samples** **Set 1 of 1**

| Channel | Ret. Time | Component Name | Area | Height | Final Amount | Final Amount Units | Peak Purity | Peak Purity Details |
|---|---|---|---|---|---|---|---|---|
| 320:10:400:10 | 3.702 | | 44681.23 | 4428.70 | | | | Not Enough Valid P |
| 282:10:400:10 | 0.629 | | 189.87 | 164.33 | | | | |
| 282:10:400:10 | 0.957 | | 4095359.46 | 1500449.48 | | | | |
| 282:10:400:10 | 1.015 | | 2130558.60 | 130531.90 | | | | |
| 282:10:400:10 | 1.028 | | 2240091.40 | 305207.55 | | | | |
| 282:10:400:10 | 2.420 | | 1524.75 | 338.90 | | | | |
| 282:10:400:10 | 3.702 | Usnic Acid | 134820.42 | 13996.50 | 10.133877 | µg/mL | | |
| 282:10:400:10 | 9.103 | | 83.57 | 120.06 | | | | |

UA 10ug rec : 282:10:400:10 : 3

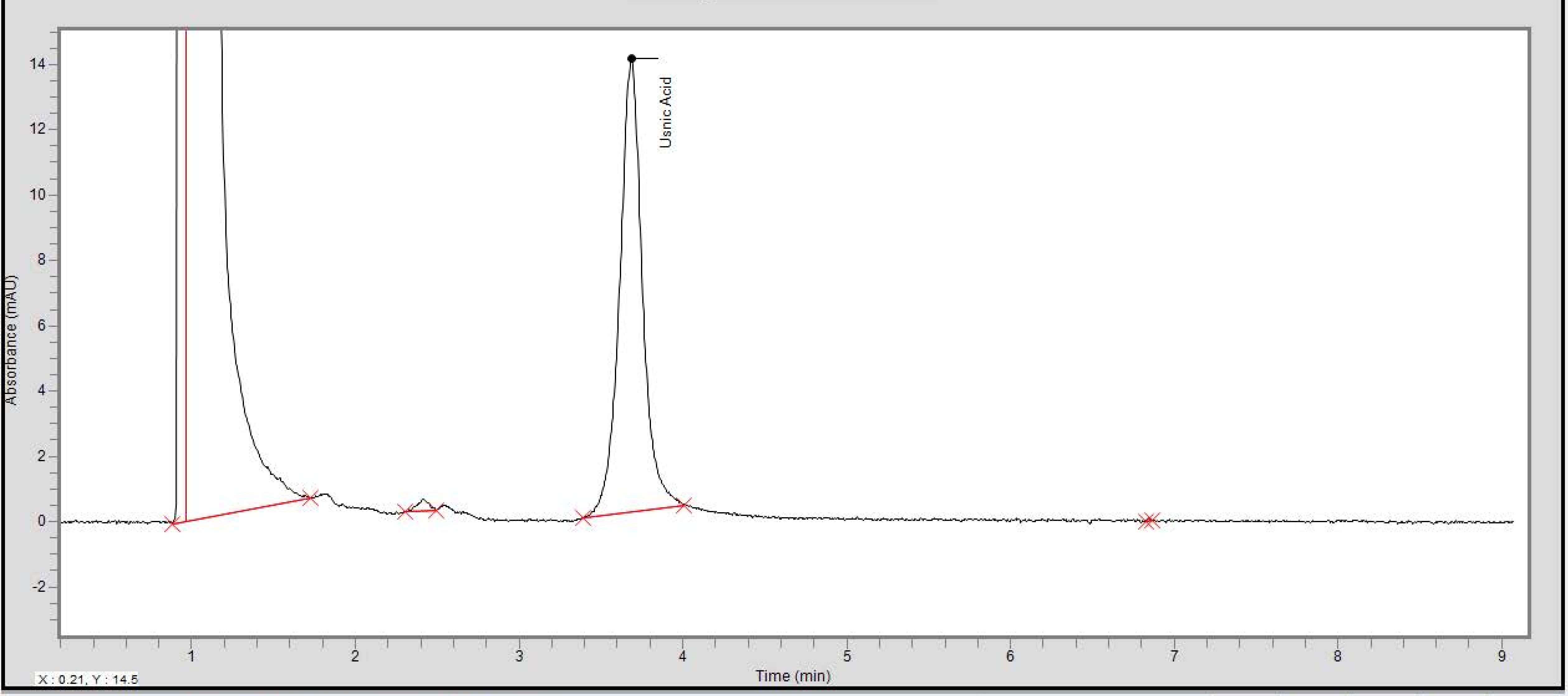


Review Calibration | Apply Calibration | Review Samples | Set 1 of 1

| Sample Name | Sample Description | Injection Number |
|---|---|---|
| UA 10ug rec | | 1 |
| UA 10ug rec | | 2 |
| UA 10ug rec | | 3 |

| Channel | Ret. Time | Component Name | Area | Height | Final Amount | Final Amount Units | Peak Purity | Peak Purity Details |
|---|---|---|---|---|---|---|---|---|
| 320:10:400:10 | 1.016 | | 1404099.51 | 302454.87 | | | 1.61 | Fail |
| 320:10:400:10 | 3.686 | | 43817.85 | 4358.06 | | | | Not Enough Valid P |
| 282:10:400:10 | 0.952 | | 7210978.79 | 2572132.55 | | | | |
| 282:10:400:10 | 0.984 | | 15581249.54 | 2599777.43 | | | | |
| 282:10:400:10 | 2.420 | | 1764.61 | 356.50 | | | | |
| 282:10:400:10 | 3.685 | Usnic Acid | 133733.29 | 13857.79 | 10.059157 | µg/mL | | |
| 282:10:400:10 | 6.842 | | 135.30 | 111.11 | | | | |

UA 10ug rec : 282:10:400:10 : 1

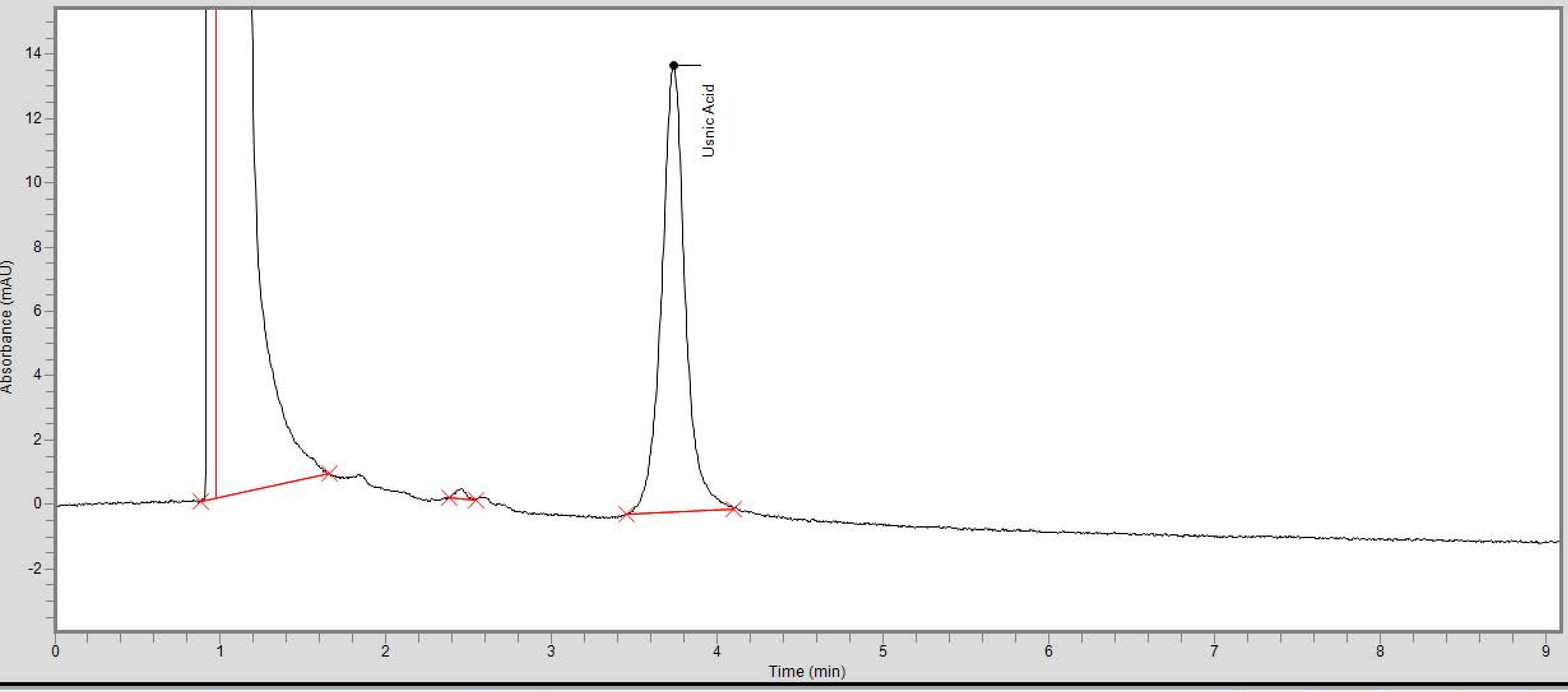


Review Calibration | Apply Calibration | Review Samples | Set 1 of 1

| Sample Name | Sample Description | Injection Number |
|---|---|---|
| UA 10ug rec | | 1 |

| Channel | Ret. Time | Component Name | Area | Height | Final Amount | Final Amount Units | Peak Purity | Peak Purity Details |
|---|---|---|---|---|---|---|---|---|
| 320:10:400:10 | 1.028 | | 1328173.39 | 271241.57 | | | 1.46 | Pass |
| 320:10:400:10 | 3.737 | | 37186.60 | 4141.71 | | | | Not Enough Valid P |
| 320:10:400:10 | 4.499 | | 52.04 | 72.01 | | | 9.96 | Fail |
| 320:10:400:10 | 6.025 | | 58.49 | 65.68 | | | 6.62 | Fail |
| 320:10:400:10 | 6.697 | | 38.45 | 46.47 | | | | Not Enough Valid P |
| 282:10:400:10 | 0.965 | | 7297121.88 | 2572304.03 | | | | |
| 282:10:400:10 | 1.023 | | 16013333.66 | 2596999.90 | | | | |
| 282:10:400:10 | 2.457 | | 1315.69 | 308.15 | | | | |
| 282:10:400:10 | 3.734 | Usnic Acid | 137647.59 | 13889.07 | 10.328192 | µg/mL | | |
