# Supplementary material for: Antioxidant, Cytotoxic, and Rheological Properties of Canola Oil Extract of Usnea barbata (L.) Weber ex F.H. Wigg from Călimani Mountains, Romania
Source: Plants (Basel). 2022 Mar 23;11(7):854. doi: 10.3390/plants11070854 (PMC9002375; doi:10.3390/plants11070854)
Supplement: Supplementary file 1 [file plants-11-00854-s001.zip › UHPLC Precision.pdf]

Sample Report - Multi-Channel

Sample Name	QC 7.5 ug/mL		
Batch Group/Name	UMF Ovidius/20211109 Usnea barbata oil extract - Copy 11-10-202		
Acquisition Date/Time	11.9.2021 10:19:54 PM		
Acquisition Method	20211103 Usnic Acid Oil		
Processing Method	20211103 Usnic Acid Oil		
Instrument Name	HPLC-PDA Plus		
Vial Number	9	Injection Number	1
Operator	dan.rambu	Chromera Version	4.2.0.6

QC 7.5 ug/mL : 320:10:400:10 : 1

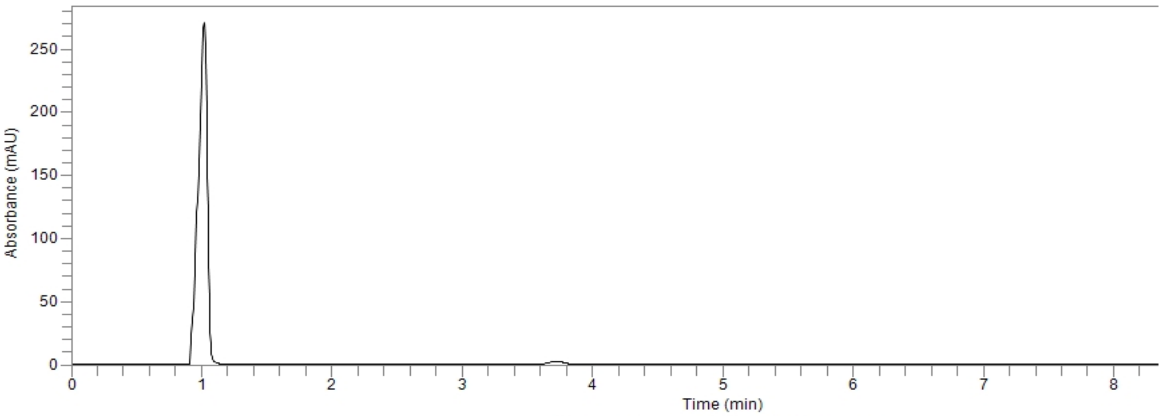

QC 7.5 ug/mL : 282:10:400:10 : 1

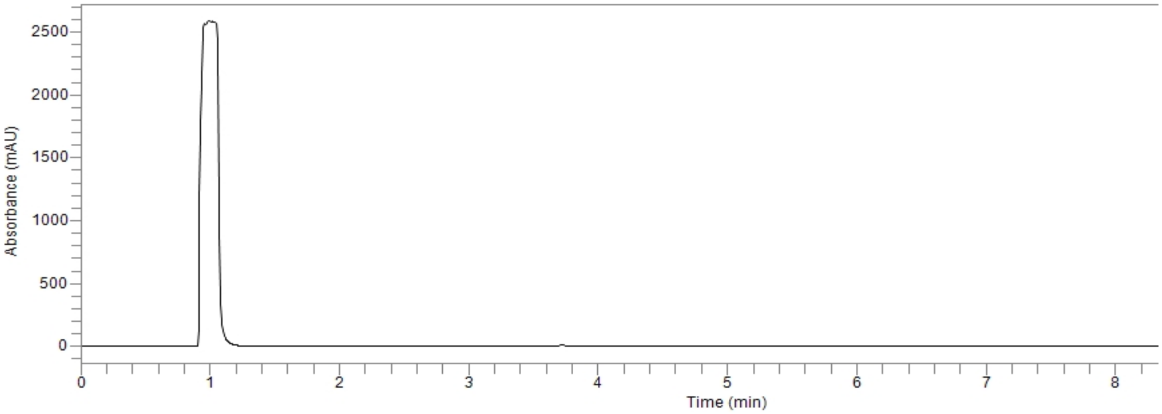

Peak #	RT (min)	Component Name	Channel	Area	Height	BL
1	0.997		282:10:400:10	22,677,961	2,590,940	BB

1	1.016		320:10:400:10	1,344,208	271,096	BB
2	3.723	Usnic Acid	282:10:400:10	88,436	8,764	BB
2	3.724		320:10:400:10	24,739	2,682	BB
**Total**				24,135,343		

1 08-56-41 - Copy 11-

415

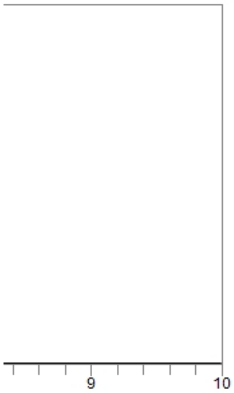

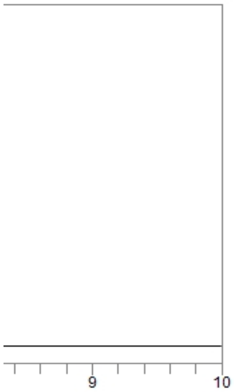

Final Amount	Units

6.9458	µg/mL
6.9458	

Sample Report - Multi-Channel

Sample Name	QC 7.5 ug/mL		
Batch Group/Name	UMF Ovidius/20211109 Usnea barbata oil extract - Copy 11-10-202		
Acquisition Date/Time	11.9.2021 10:54:58 PM		
Acquisition Method	20211103 Usnic Acid Oil		
Processing Method	20211103 Usnic Acid Oil		
Instrument Name	HPLC-PDA Plus		
Vial Number	9	Injection Number	4
Operator	dan.rambu	Chromera Version	4.2.0.6

QC 7.5 ug/mL : 320:10:400:10 : 4

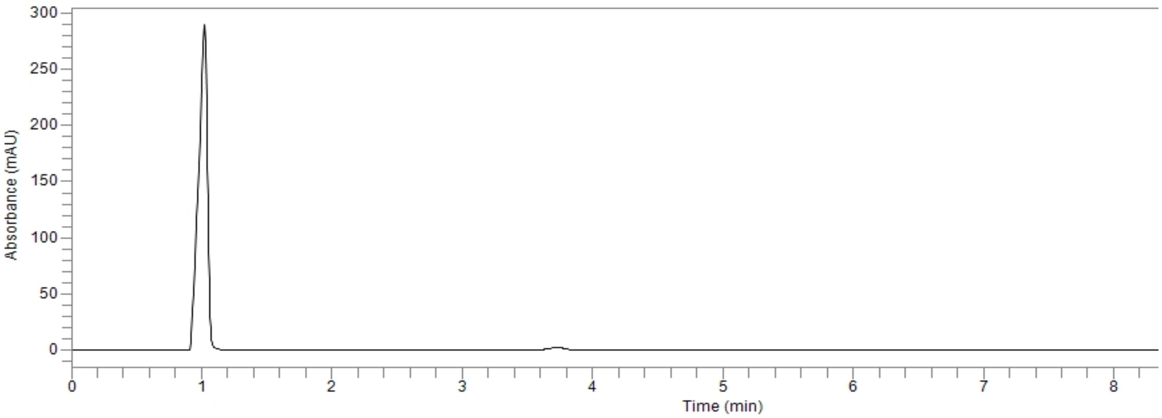

QC 7.5 ug/mL : 282:10:400:10 : 4

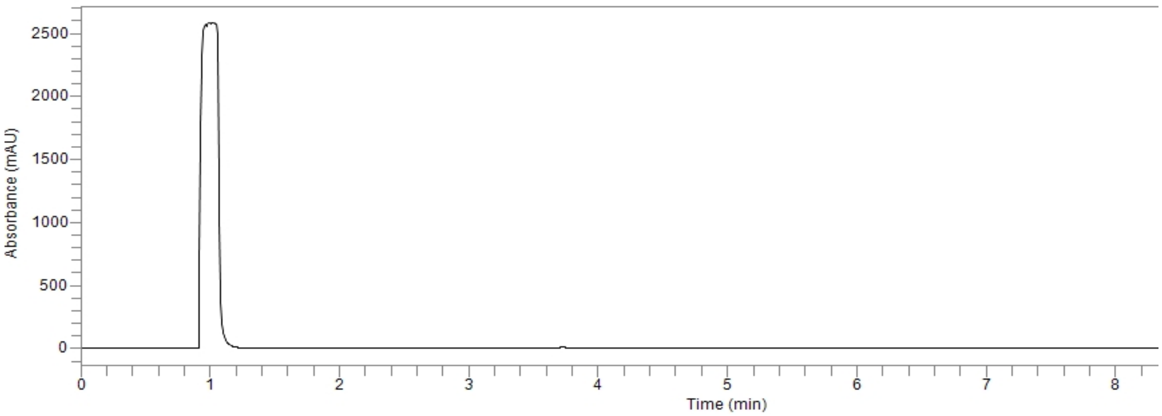

Peak #	RT (min)	Component Name	Channel	Area	Height	BL
1	0.995		282:10:400:10	22,603,492	2,585,938	BB

1	1.019		320:10:400:10	1,405,142	290,302	BB
2	3.727		320:10:400:10	27,012	2,765	BB
2	3.728	Usnic Acid	282:10:400:10	92,735	8,982	BB
**Total**				24,128,381		

1 08-56-41 - Copy 11-

415

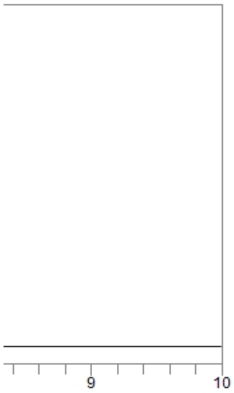

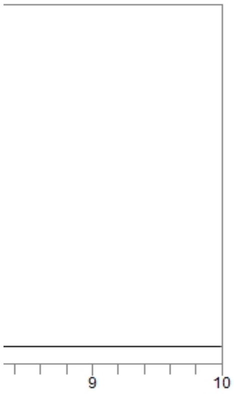

Final Amount	Units

7.2413	µg/mL
7.2413	

Sample Report - Multi-Channel

Sample Name	QC 7.5 ug/mL		
Batch Group/Name	UMF Ovidius/20211109 Usnea barbata oil extract - Copy 11-10-202		
Acquisition Date/Time	11.9.2021 11:06:39 PM		
Acquisition Method	20211103 Usnic Acid Oil		
Processing Method	20211103 Usnic Acid Oil		
Instrument Name	HPLC-PDA Plus		
Vial Number	9	Injection Number	5
Operator	dan.rambu	Chromera Version	4.2.0.6

QC 7.5 ug/mL : 320:10:400:10 : 5

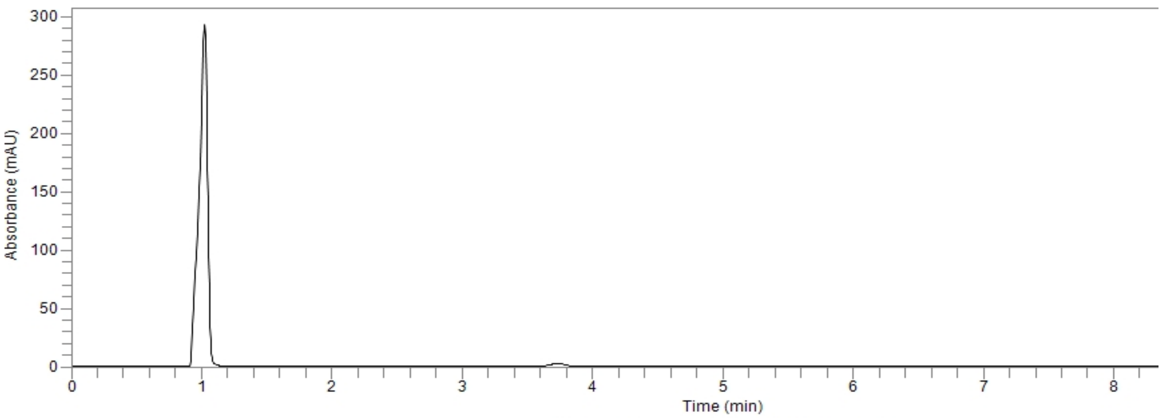

QC 7.5 ug/mL : 282:10:400:10 : 5

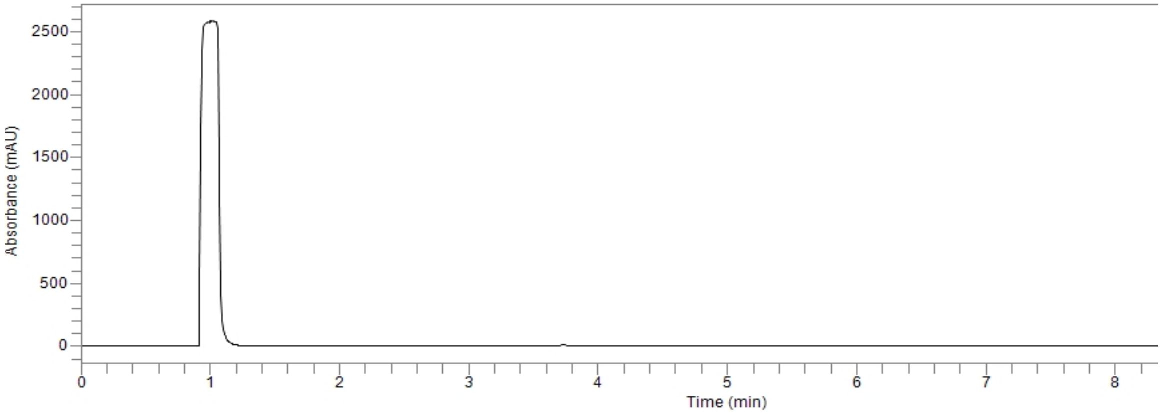

Peak #	RT (min)	Component Name	Channel	Area	Height	BL
1	1.020		320:10:400:10	1,376,726	293,043	BB

1	1.021		282:10:400:10	22,656,659	2,589,605	BB
2	3.736	Usnic Acid	282:10:400:10	91,150	8,897	BB
2	3.740		320:10:400:10	25,681	2,710	BB
**Total**				24,150,217		

1 08-56-41 - Copy 11-

415

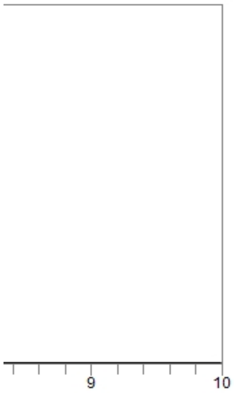

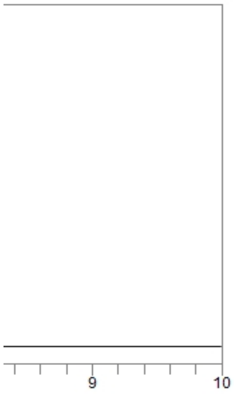

Final Amount	Units

7.1324	µg/mL
7.1324	

Sample Report - Multi-Channel

Sample Name	QC 7.5 ug/mL		
Batch Group/Name	UMF Ovidius/20211109 Usnea barbata oil extract - Copy 11-10-202		
Acquisition Date/Time	11.9.2021 11:18:20 PM		
Acquisition Method	20211103 Usnic Acid Oil		
Processing Method	20211103 Usnic Acid Oil		
Instrument Name	HPLC-PDA Plus		
Vial Number	9	Injection Number	6
Operator	dan.rambu	Chromera Version	4.2.0.6[illegible]

QC 7.5 ug/mL : 320:10:400:10 : 6

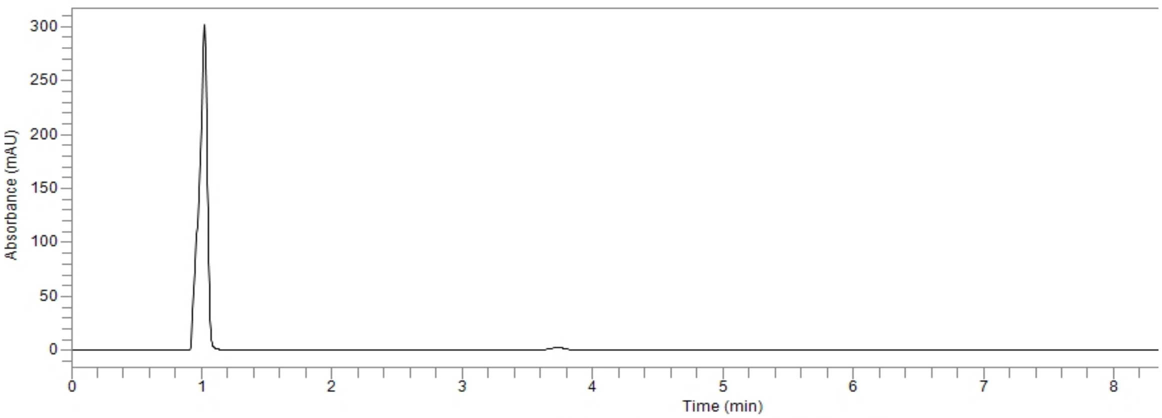

QC 7.5 ug/mL : 282:10:400:10 : 6

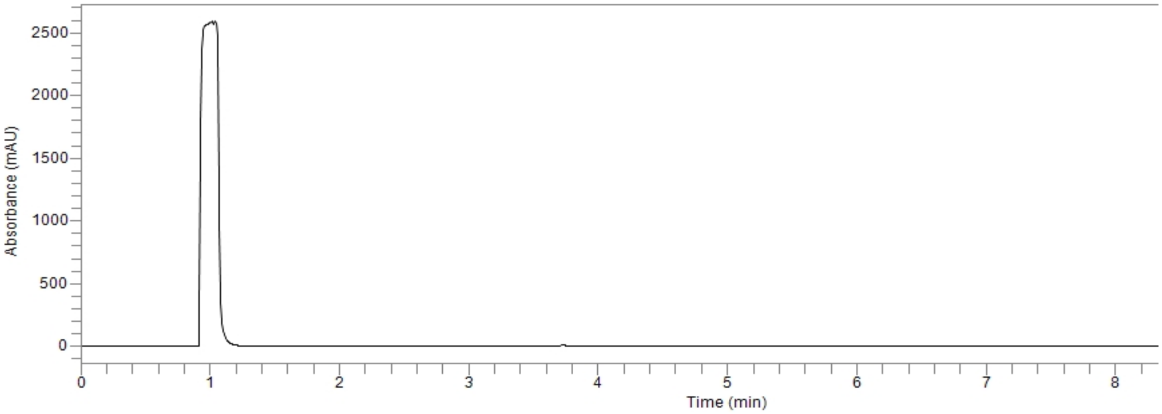

Peak #	RT (min)	Component Name	Channel	Area	Height	BL
1	1.013		282:10:400:10	22,336,008	2,594,293	BB

1	1.018		320:10:400:10	1,349,530	302,955	BB
2	3.732	Usnic Acid	282:10:400:10	89,034	8,775	BB
2	3.734		320:10:400:10	24,395	2,653	BB
**Total**				23,798,967		

1 08-56-41 - Copy 11-

415

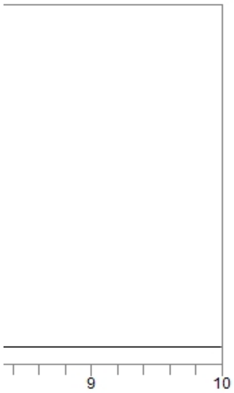

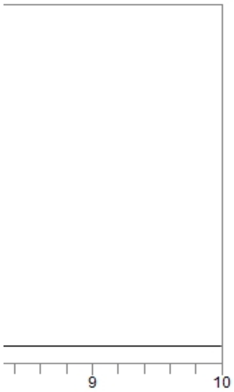

Final Amount	Units

6.9869	µg/mL
6.9869	

Sample Report - Multi-Channel

Sample Name	QC 7.5 ug/mL		
Batch Group/Name	UMF Ovidius/20211109 Usnea barbata oil extract - Copy 11-10-202		
Acquisition Date/Time	11.9.2021 10:31:36 PM		
Acquisition Method	20211103 Usnic Acid Oil		
Processing Method	20211103 Usnic Acid Oil		
Instrument Name	HPLC-PDA Plus		
Vial Number	9	Injection Number	2
Operator	dan.rambu	Chromera Version	4.2.0.6[illegible]

QC 7.5 ug/mL : 320:10:400:10 : 2

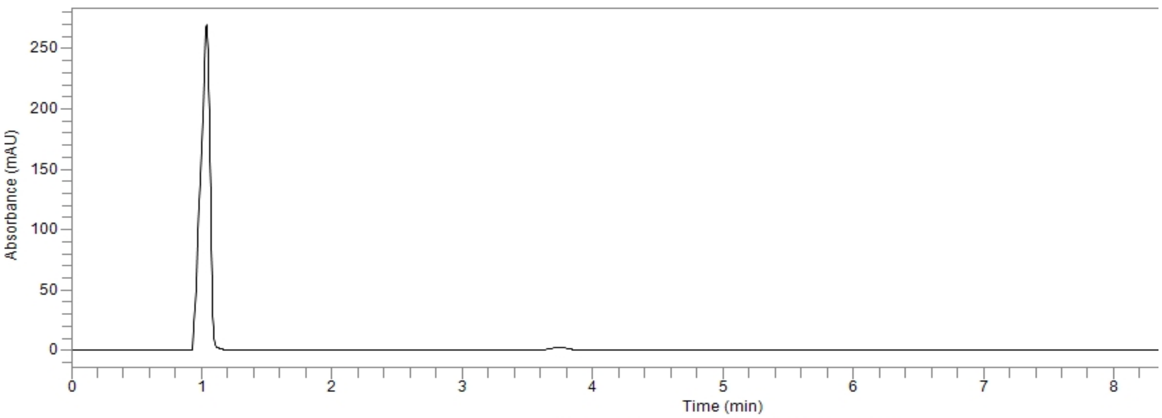

QC 7.5 ug/mL : 282:10:400:10 : 2

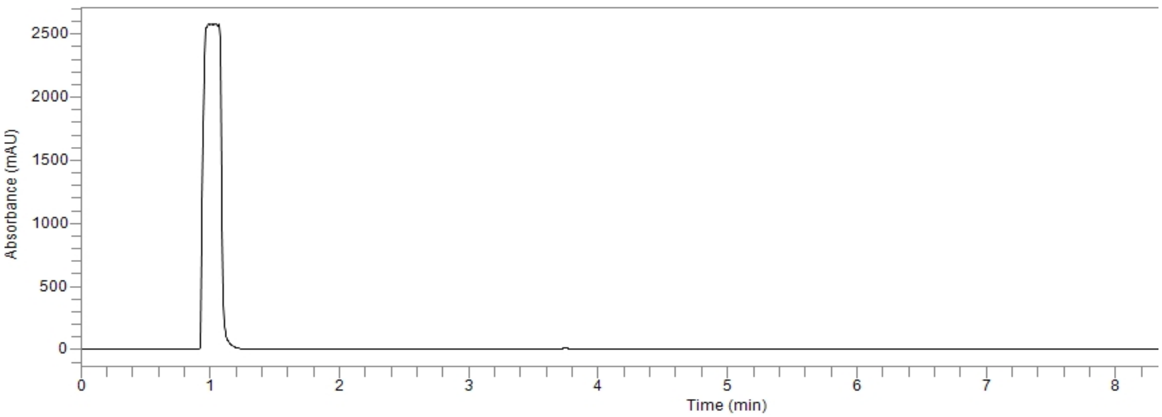

Peak #	RT (min)	Component Name	Channel	Area	Height	BL
1	1.037		320:10:400:10	1,398,859	270,477	BB

1	1.042		282:10:400:10	22,904,320	2,584,995	BB
2	3.749	Usnic Acid	282:10:400:10	90,957	8,901	BB
2	3.750		320:10:400:10	25,569	2,735	BB
**Total**				24,419,705		

1 08-56-41 - Copy 11-

415

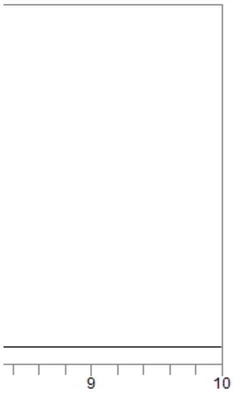

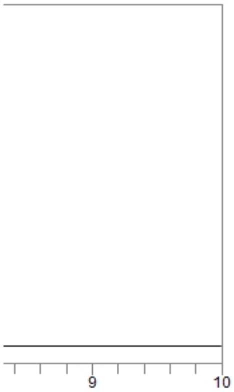

Final Amount	Units

7.1191	µg/mL
7.1191	

Sample Report - Multi-Channel

Sample Name	QC 7.5 ug/mL		
Batch Group/Name	UMF Ovidius/20211109 Usnea barbata oil extract - Copy 11-10-202		
Acquisition Date/Time	11.9.2021 10:43:17 PM		
Acquisition Method	20211103 Usnic Acid Oil		
Processing Method	20211103 Usnic Acid Oil		
Instrument Name	HPLC-PDA Plus		
Vial Number	9	Injection Number	3
Operator	dan.rambu	Chromera Version	4.2.0.6[illegible]

QC 7.5 ug/mL : 320:10:400:10 : 3

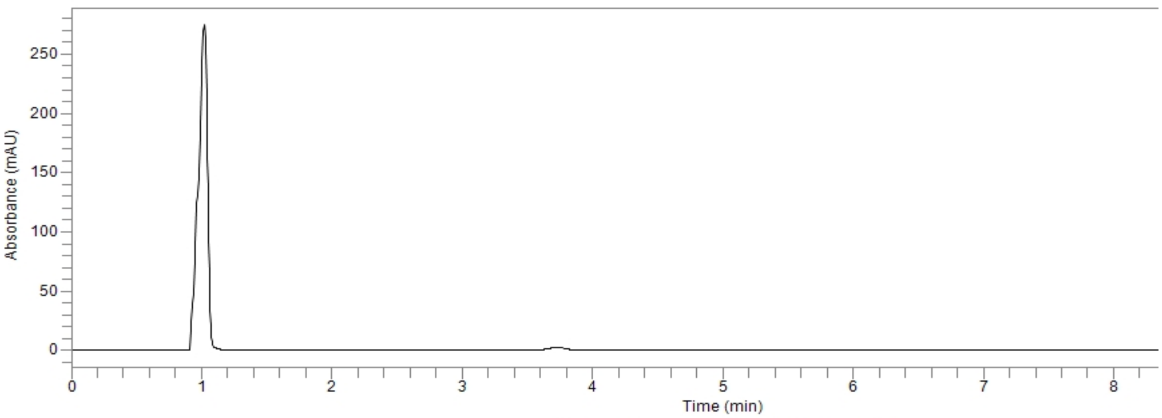

QC 7.5 ug/mL : 282:10:400:10 : 3

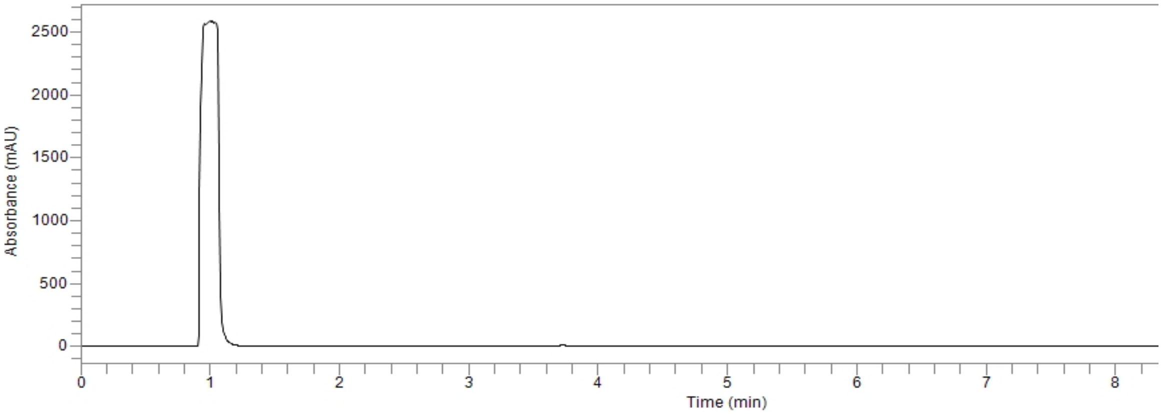

Peak #	RT (min)	Component Name	Channel	Area	Height	BL
1	1.014		282:10:400:10	23,080,954	2,592,497	BB

1	1.019		320:10:400:10	1,388,632	274,288	BB
2	3.729	Usnic Acid	282:10:400:10	91,862	8,883	BB
2	3.731		320:10:400:10	25,149	2,701	BB
**Total**				24,586,598		

1 08-56-41 - Copy 11-

415

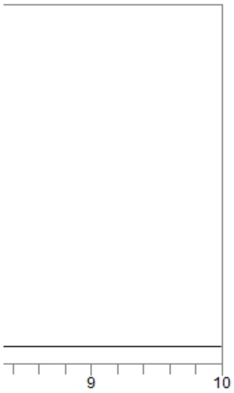

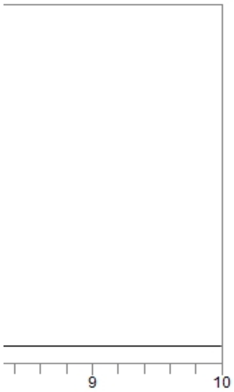

Final Amount	Units

7.1813	µg/mL
7.1813	

Sample Report - Multi-Channel

Sample Name	UBO 10 mg/ML		
Batch Group/Name	UMF Ovidius/20211109 Usnea barbata oil extract - Copy 11-10-202		
Acquisition Date/Time	11.9.2021 11:30:01 PM		
Acquisition Method	20211103 Usnic Acid Oil		
Processing Method	20211103 Usnic Acid Oil		
Instrument Name	HPLC-PDA Plus		
Vial Number	10	Injection Number	1
Operator	dan.rambu	Chromera Version	4.2.0.6[illegible]

UBO 10 mg/ML : 320:10:400:10 : 1

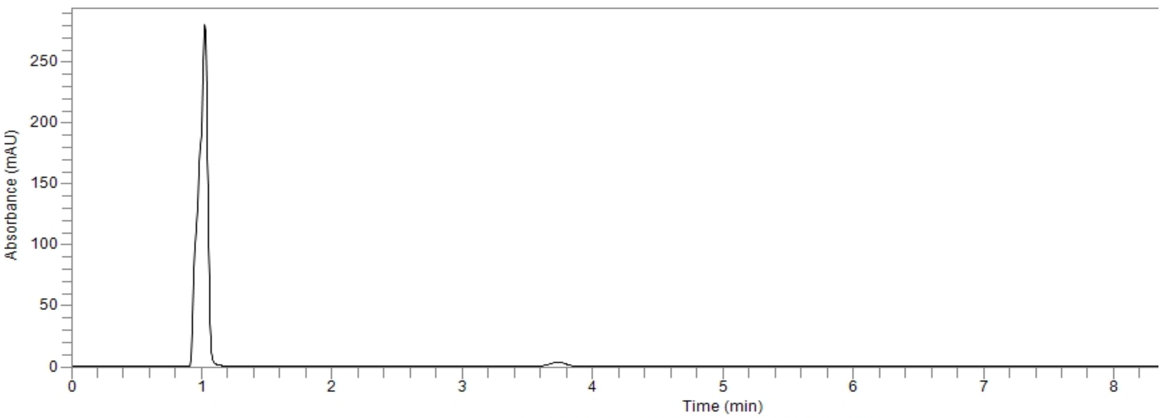

UBO 10 mg/ML : 282:10:400:10 : 1

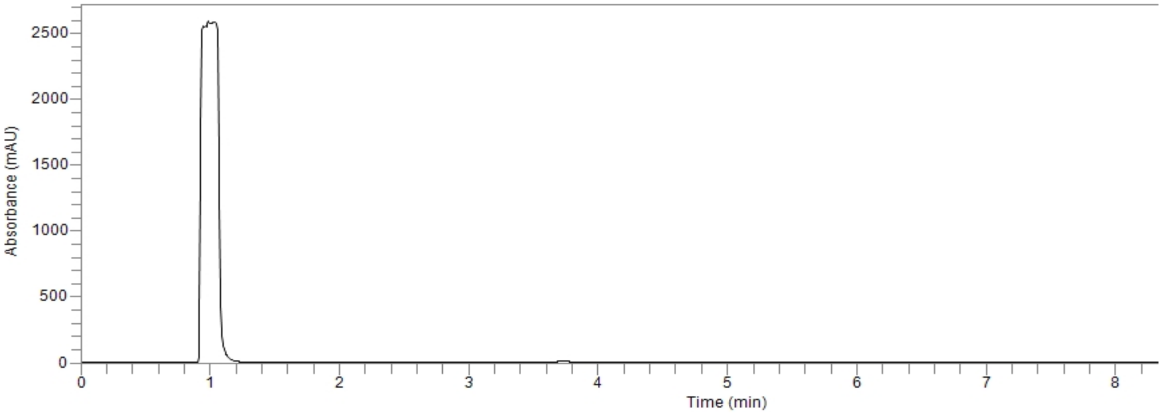

Peak #	RT (min)	Component Name	Channel	Area	Height	BL
1	1.022		320:10:400:10	1,394,209	280,739	BB

1	1.040		282:10:400:10	22,857,228	2,589,158	BE
2	1.845		282:10:400:10	30,779	1,553	EV
3	2.450		282:10:400:10	11,736	1,338	VV
4	2.585		282:10:400:10	8,724	910	VB
5	3.735	Usnic Acid	282:10:400:10	130,381	12,729	BB
2	3.736		320:10:400:10	38,721	3,945	BB
**Total**				24,471,778		

1 08-56-41 - Copy 11-

415

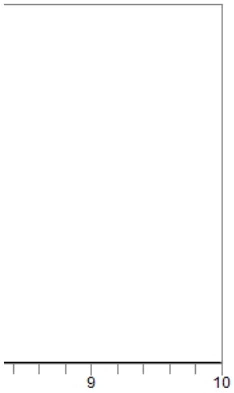

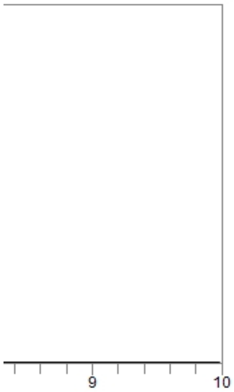

Final Amount	Units

9.8288	µg/mL
9.8288	

Sample Report - Multi-Channel

Sample Name	UBO 10 mg/ML
Batch Group/Name	UMF Ovidius/20211109 Usnea barbata oil extract - Copy 11-10-202

Acquisition Date/Time	11.9.2021 11:41:43 PM		
Acquisition Method	20211103 Usnic Acid Oil		
Processing Method	20211103 Usnic Acid Oil		
Instrument Name	HPLC-PDA Plus		
Vial Number	10	Injection Number	2
Operator	dan.rambu	Chromera Version	4.2.0.6[illegible]

UBO 10 mg/ML : 320:10:400:10 : 2

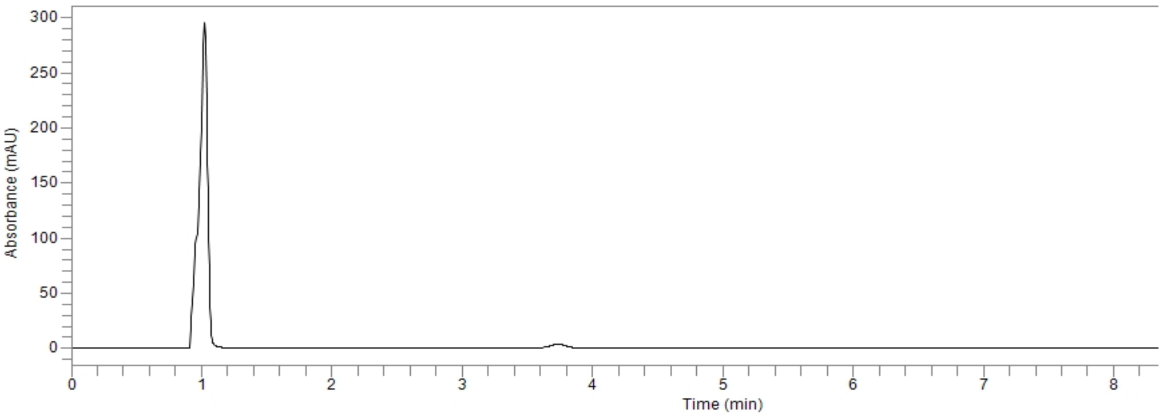

UBO 10 mg/ML : 282:10:400:10 : 2

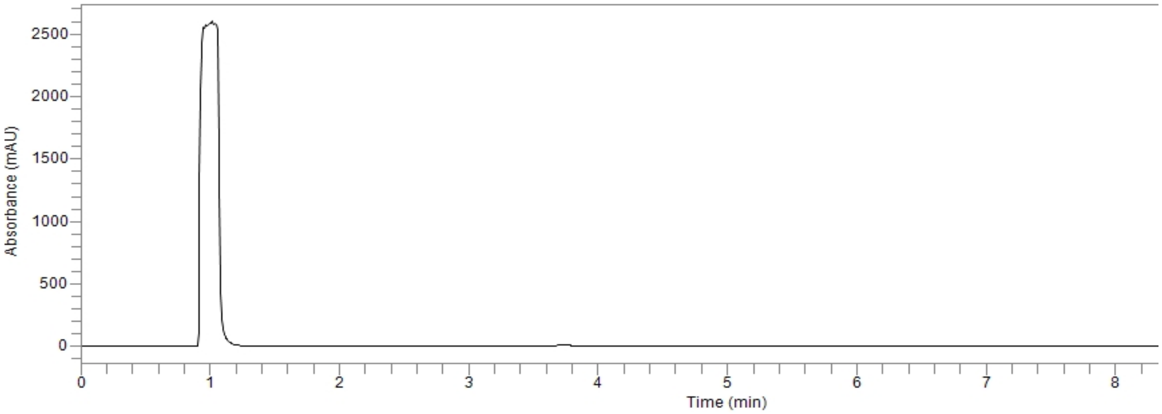

Peak #	RT (min)	Component Name	Channel	Area	Height	BL
1	1.014		282:10:400:10	23,552,505	2,605,462	BE

1	1.019		320:10:400:10	1,379,523	296,221	BB
2	1.832		320:10:400:10	353	100	BB
2	1.838		282:10:400:10	22,441	1,327	EB
3	2.452		282:10:400:10	6,421	982	BV
4	2.573		282:10:400:10	2,891	525	VB
5	3.736	Usnic Acid	282:10:400:10	132,381	12,733	BB
3	3.736		320:10:400:10	36,977	3,899	BB
**Total**				25,133,492		

1 08-56-41 - Copy 11-

415

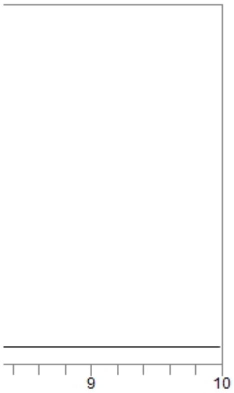

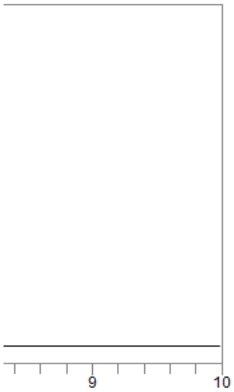

Final Amount	Units

9,9662	µg/mL
9,9662	

Sample Report - Multi-Channel

Sample Name	UBO 10 mg/ML		
Batch Group/Name	UMF Ovidius/20211109 Usnea barbata oil extract - Copy 11-10-202		
Acquisition Date/Time	11.9.2021 11:53:24 PM		
Acquisition Method	20211103 Usnic Acid Oil		
Processing Method	20211103 Usnic Acid Oil		
Instrument Name	HPLC-PDA Plus		
Vial Number	10	Injection Number	3
Operator	dan.rambu	Chromera Version	4.2.0.6[illegible]

UBO 10 mg/ML : 320:10:400:10 : 3

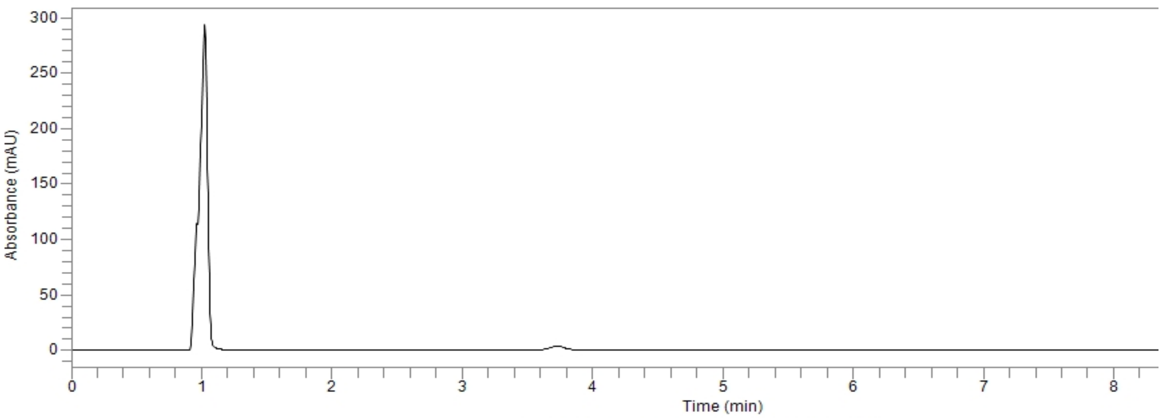

UBO 10 mg/ML : 282:10:400:10 : 3

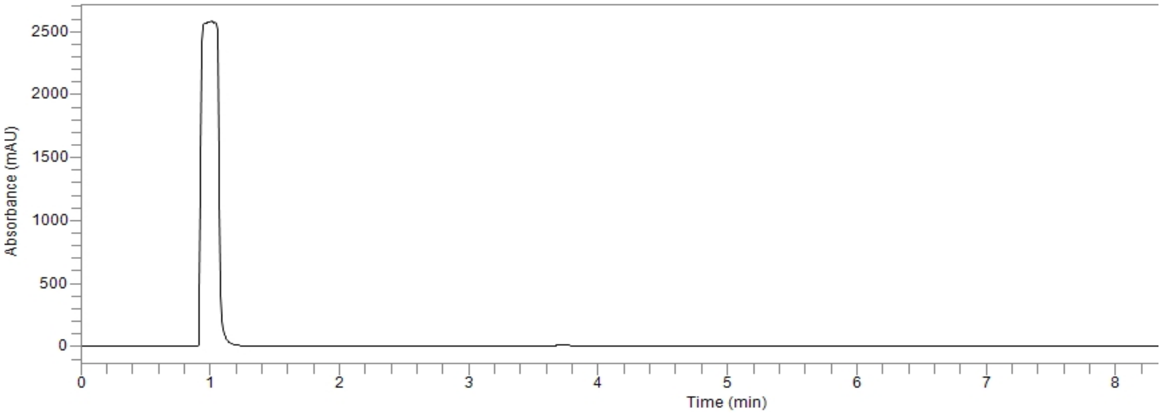

Peak #	RT (min)	Component Name	Channel	Area	Height	BL
1	1.006		282:10:400:10	22,503,179	2,586,849	BE

1	1.020		320:10:400:10	1,353,588	294,104	BB
2	1.848		282:10:400:10	14,391	1,108	EB
3	2.448		282:10:400:10	8,954	1,143	BV
4	2.575		282:10:400:10	7,539	816	VB
5	3.732	Usnic Acid	282:10:400:10	121,871	12,510	BB
2	3.734		320:10:400:10	36,101	3,819	BB
**Total**				24,045,622		

1 08-56-41 - Copy 11-

415

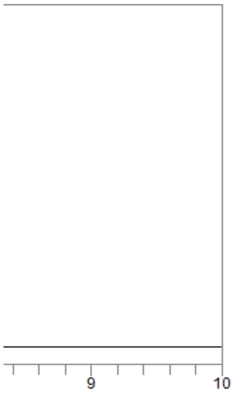

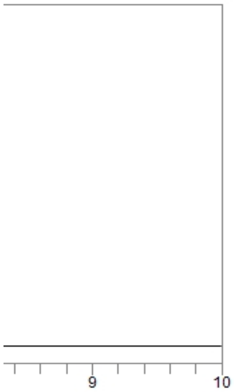

Final Amount	Units

9,2438	µg/mL
9.2438	

Sample Report - Multi-Channel

Sample Name	UBO 10 mg/ML
Batch Group/Name	UMF Ovidius/20211109 Usnea barbata oil extract - Copy 11-10-202

Acquisition Date/Time	11.10.2021 12:05:05 AM		
Acquisition Method	20211103 Usnic Acid Oil		
Processing Method	20211103 Usnic Acid Oil		
Instrument Name	HPLC-PDA Plus		
Vial Number	10	Injection Number	4
Operator	dan.rambu	Chromera Version	4.2.0.6[illegible]

UBO 10 mg/ML : 320:10:400:10 : 4

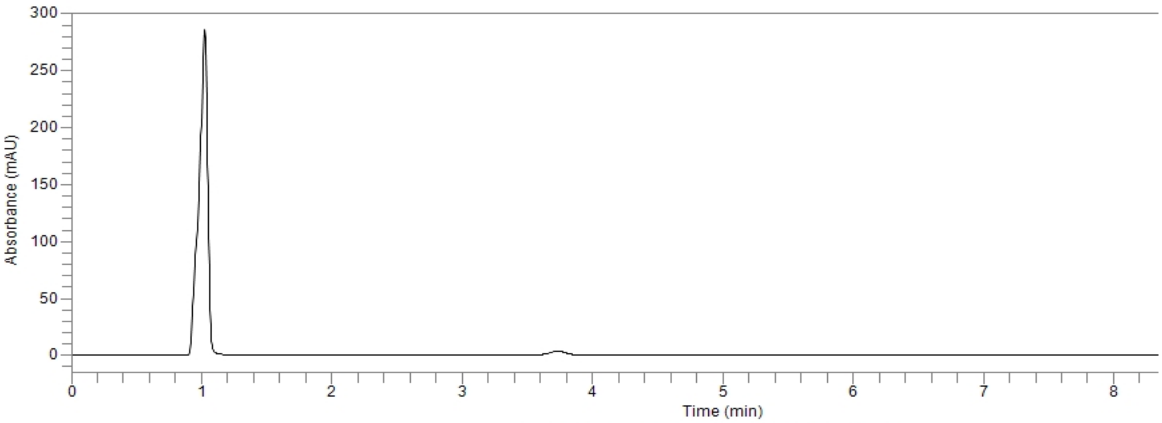

UBO 10 mg/ML : 282:10:400:10 : 4

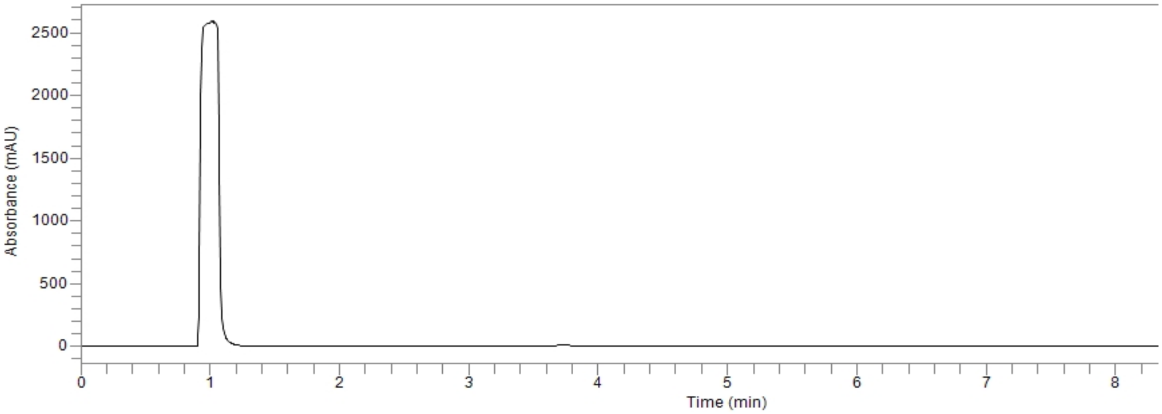

Peak #	RT (min)	Component Name	Channel	Area	Height	BL
1	1.020		320:10:400:10	1,373,874	286,886	BB

1	1.022		282:10:400:10	23,318,679	2,595,674	BE
2	1.843		282:10:400:10	31,803	1,526	EV
3	2.444		282:10:400:10	12,268	1,307	VV
4	2.580		282:10:400:10	8,966	928	VB
2	3.733		320:10:400:10	37,174	3,872	BB
5	3.734	Usnic Acid	282:10:400:10	128,650	12,620	BB
**Total**				24,911,414		

1 08-56-41 - Copy 11-

415

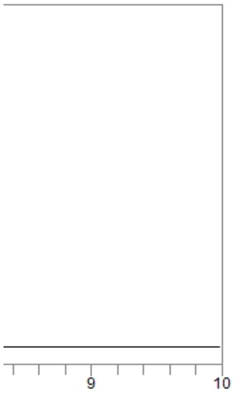

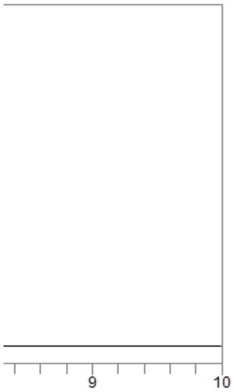

Final Amount	Units

9,7097	µg/mL
9.7097	

Sample Report - Multi-Channel

Sample Name	UBO 10 mg/ML
Batch Group/Name	UMF Ovidius/20211109 Usnea barbata oil extract - Copy 11-10-202

Acquisition Date/Time	11.10.2021 12:16:46 AM		
Acquisition Method	20211103 Usnic Acid Oil		
Processing Method	20211103 Usnic Acid Oil		
Instrument Name	HPLC-PDA Plus		
Vial Number	10	Injection Number	5
Operator	dan.rambu	Chromera Version	4.2.0.6[illegible]

UBO 10 mg/ML : 320:10:400:10 : 5

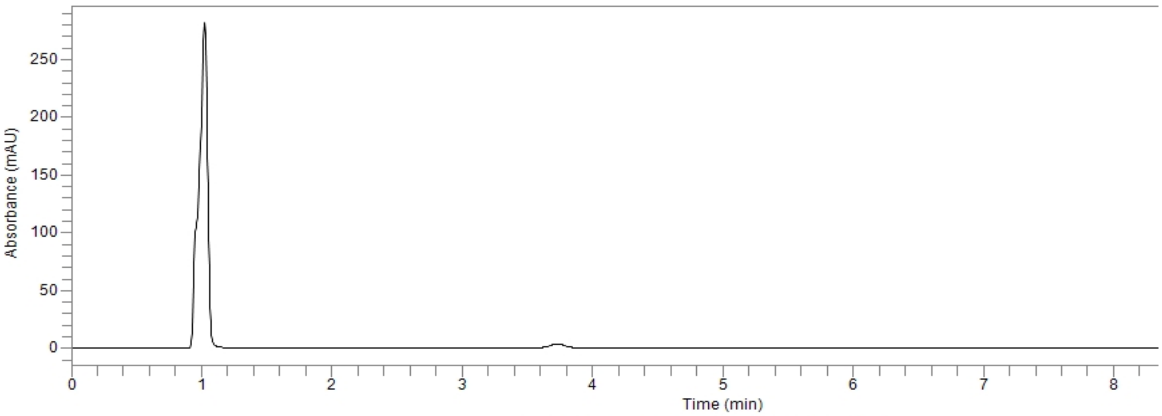

UBO 10 mg/ML : 282:10:400:10 : 5

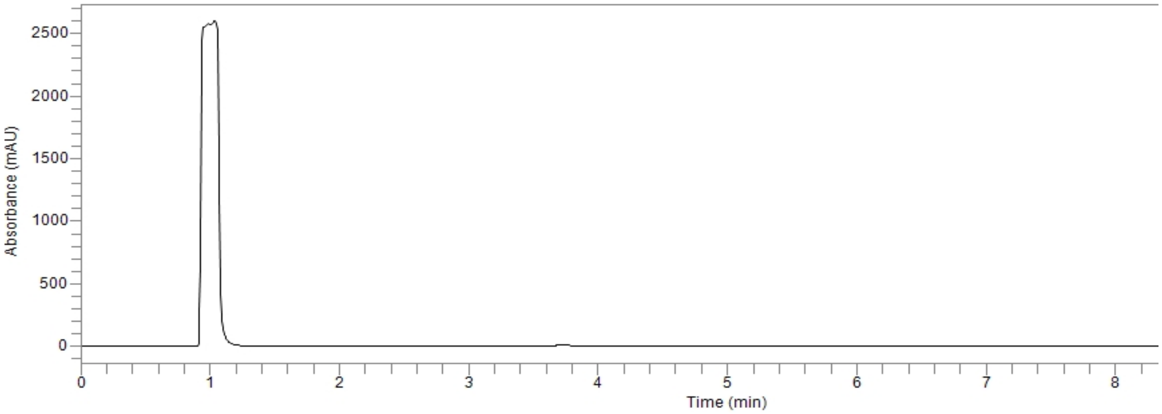

Peak #	RT (min)	Component Name	Channel	Area	Height	BL
1	1.020		320:10:400:10	1,348,971	282,618	BB

1	1.034		282:10:400:10	22,161,319	2,604,552	BE
2	1.840		282:10:400:10	28,940	1,528	EV
3	2.450		282:10:400:10	12,313	1,309	VV
4	2.577		282:10:400:10	9,212	942	VB
2	3.731		320:10:400:10	36,739	3,862	BB
5	3.731	Usnic Acid	282:10:400:10	129,583	12,595	BB
**Total**				23,727,078		

1 08-56-41 - Copy 11-

415

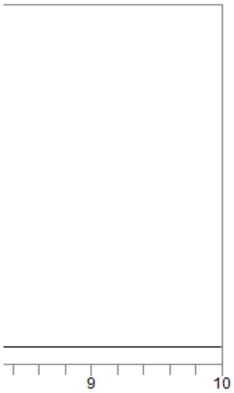

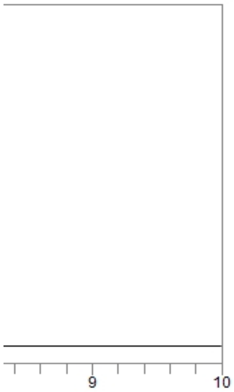

Final Amount	Units

9,7739	µg/mL
9.7739	

Sample Report - Multi-Channel

Sample Name	UBO 10 mg/ML		
Batch Group/Name	UMF Ovidius/20211109 Usnea barbata oil extract - Copy 11-10-202		
Acquisition Date/Time	11.10.2021 12:28:26 AM		
Acquisition Method	20211103 Usnic Acid Oil		
Processing Method	20211103 Usnic Acid Oil		
Instrument Name	HPLC-PDA Plus		
Vial Number	10	Injection Number	6
Operator	dan.rambu	Chromera Version	4.2.0.6

UBO 10 mg/ML : 320:10:400:10 : 6

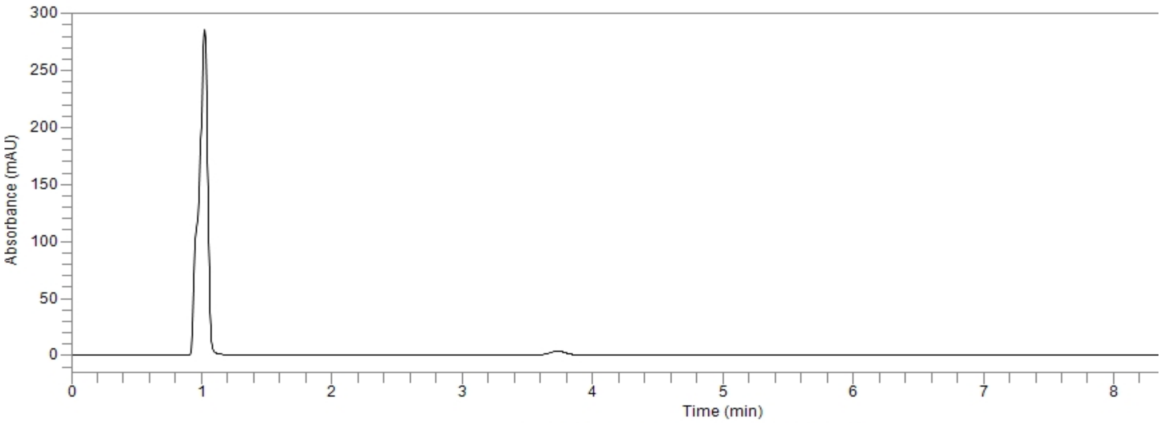

UBO 10 mg/ML : 282:10:400:10 : 6

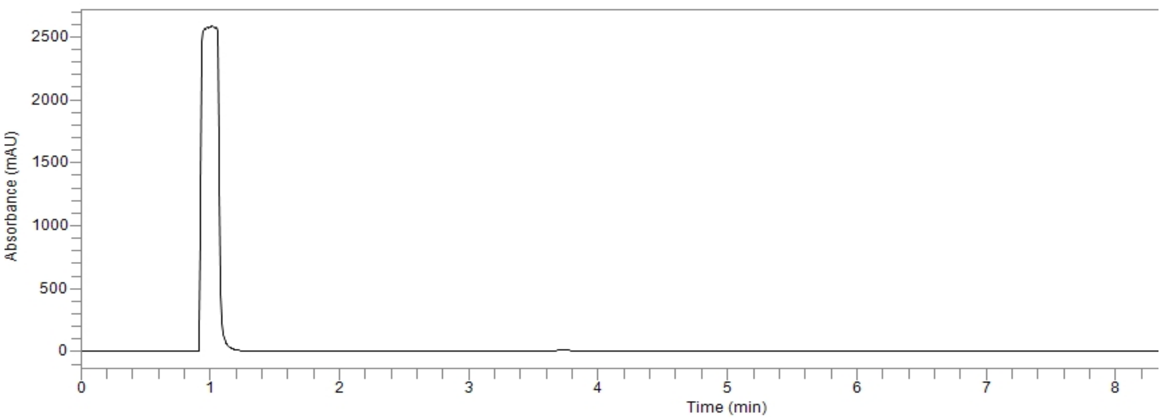

Peak #	RT (min)	Component Name	Channel	Area	Height	BL
1	1.011		282:10:400:10	22,466,679	2,588,742	BE

1	1.020		320:10:400:10	1,381,836	286,639	BB
2	1.842		282:10:400:10	33,114	1,573	EV
3	2.449		282:10:400:10	12,483	1,335	VV
4	2.580		282:10:400:10	8,557	933	VB
2	3.733		320:10:400:10	37,959	3,918	BB
5	3.734	Usnic Acid	282:10:400:10	133,576	12,762	BB
**Total**				24,074,206		

1 08-56-41 - Copy 11-

415

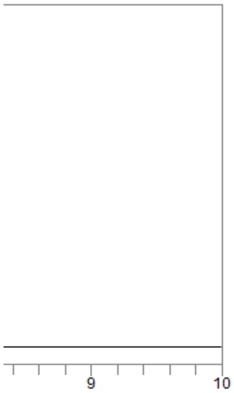

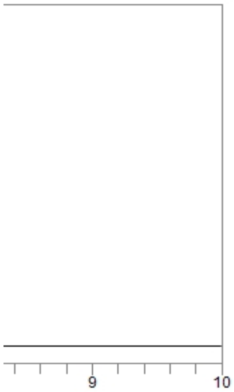

Final Amount	Units

10,0484	µg/mL
10.0484	
