# Supplementary material for: Antioxidant, Cytotoxic, and Rheological Properties of Canola Oil Extract of Usnea barbata (L.) Weber ex F.H. Wigg from Călimani Mountains, Romania
Source: Plants (Basel). 2022 Mar 23;11(7):854. doi: 10.3390/plants11070854 (PMC9002375; doi:10.3390/plants11070854)
Supplement: Supplementary file 1 [file plants-11-00854-s001.zip › UHPLC Linearity.pdf]

Sample Replicate Report - Multi-Channel

Sample Name	UA std 2.5 ug/mL		
Batch Group/Name	UMF Ovidius/20211109 Usnea barbata oil extract - Copy 11-10-2021 08-56-41 - Copy 11-10-2021 09-15-52 - [illegible]		
Acquisition Method	20211103 Usnic Acid Oil		
Processing Method	20211103 Usnic Acid Oil		
Instrument Name	HPLC-PDA Plus		
Vial Number	4	Operator	dan.rambu
Acquisition Date	11/9/2021	Chromera Version	4.2.0.6415

UA std 2.5 ug/mL : 320:10:400:10 : 1

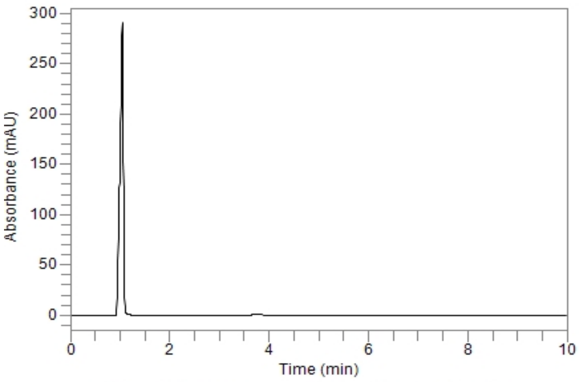

UA std 2.5 ug/mL : 320:10:400:10 : 2

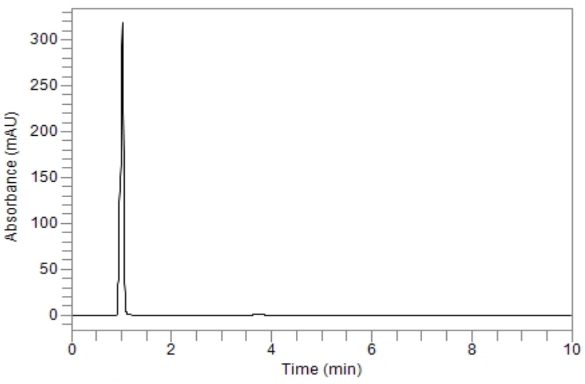

UA std 2.5 ug/mL : 320:10:400:10 : 3

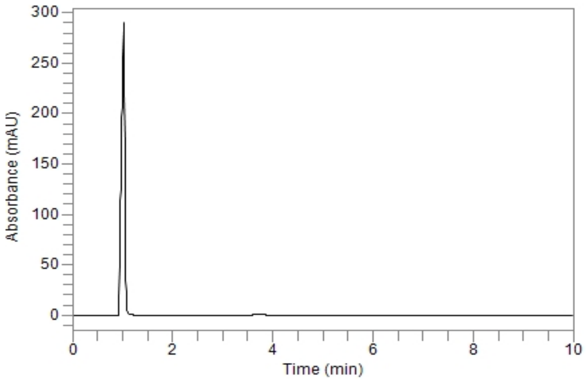

UA std 2.5 ug/mL : 320:10:400:10 : 4

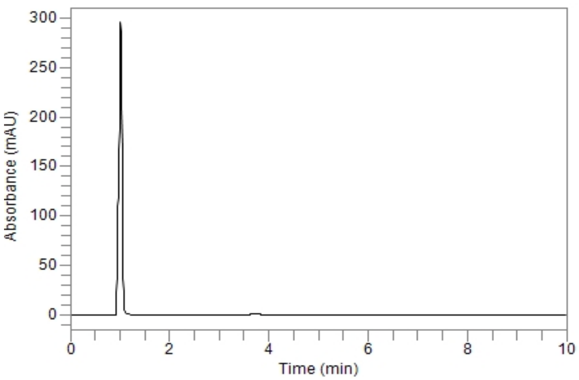

UA std 2.5 ug/mL : 320:10:400:10 : 5

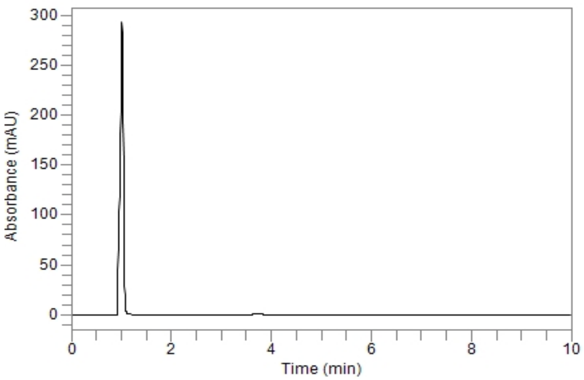

Channel Name **320:10:400:10**

Component Name	Avg RT (min)	RT %RSD	Avg Area	Area %RSD	Avg Height	Height %RSD	Avg Final Amount	Final %RSD
	N/A	N/A	N/A	N/A	N/A	N/A	N/A	N/A

UA std 2.5 ug/mL : 282:10:400:10 : 1

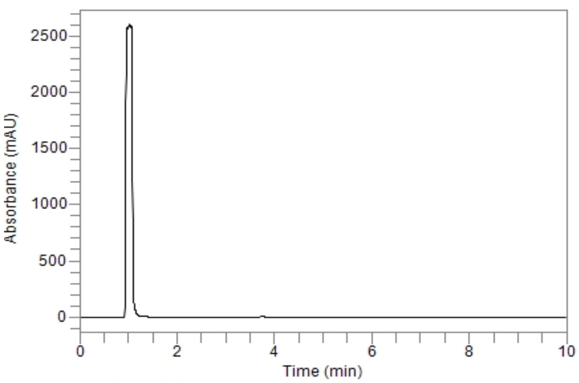

UA std 2.5 ug/mL : 282:10:400:10 : 2

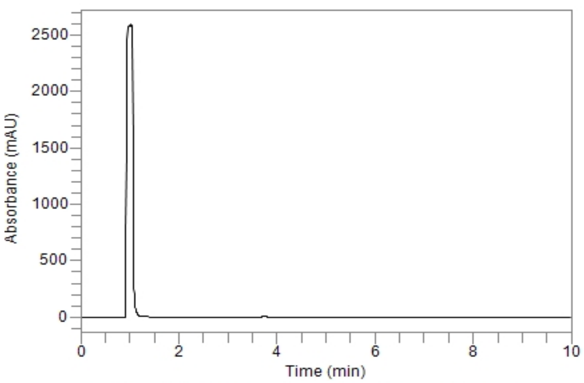

UA std 2.5 ug/mL : 282:10:400:10 : 3

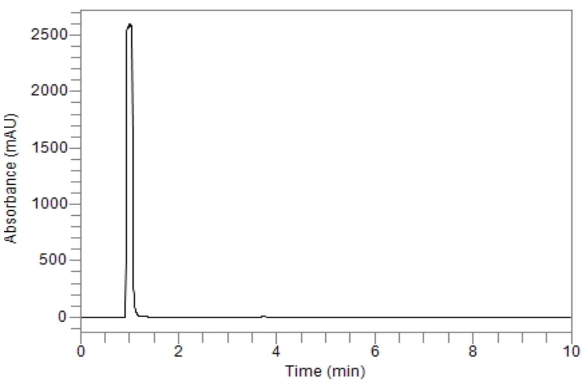

UA std 2.5 ug/mL : 282:10:400:10 : 4

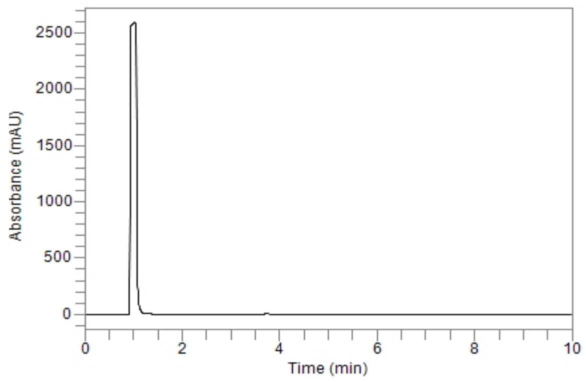

UA std 2.5 ug/mL : 282:10:400:10 : 5

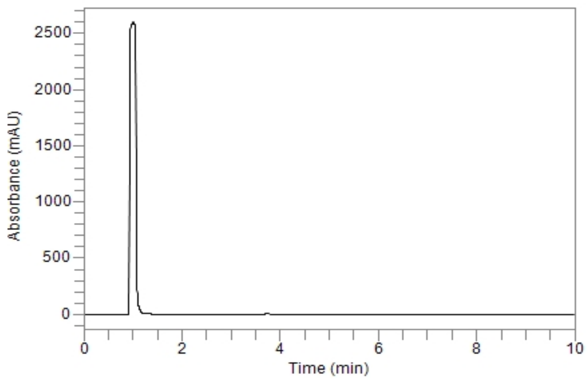

Channel Name **282:10:400:10**

Component Name	Avg RT (min)	RT %RSD	Avg Area	Area %RSD	Avg Height	Height %RSD	Avg Final Amount	Final %RSD
Usnic Acid	3.741	0.284	34,307.6	1.465	3,508.7	0.547	3.2255	1.071

Copy 11-10-

Sample Replicate Report - Multi-Channel

Sample Name	UA std 5 ug/mL		
Batch Group/Name	UMF Ovidius/20211109 Usnea barbata oil extract - Copy 11-10-2021 08-56-41 - Copy 11-10-2021 09-15-52 - (		
Acquisition Method	20211103 Usnic Acid Oil		
Processing Method	20211103 Usnic Acid Oil		
Instrument Name	HPLC-PDA Plus		
Vial Number	5	Operator	dan.rambu
Acquisition Date	11/9/2021	Chromera Version	4.2.0.6415

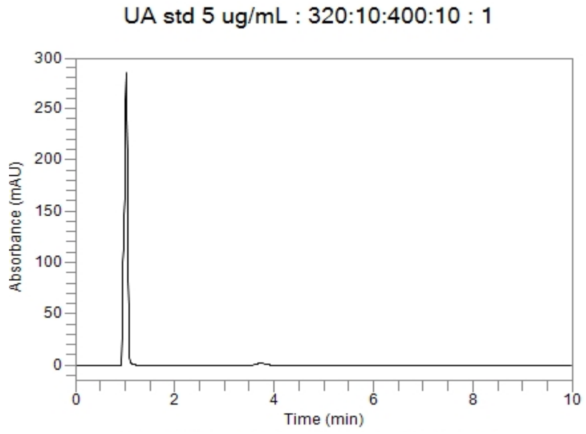

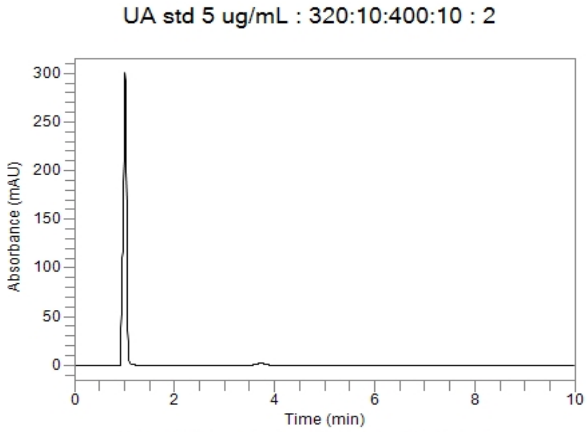

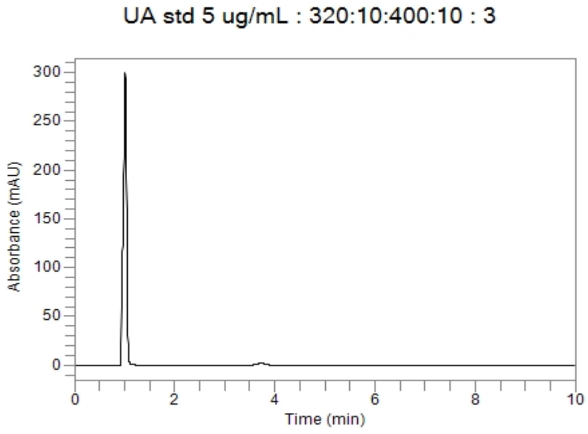

UA std 5 ug/mL : 320:10:400:10 : 4

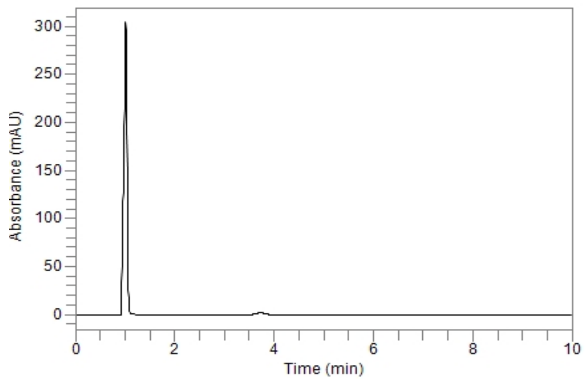

UA std 5 ug/mL : 320:10:400:10 : 5

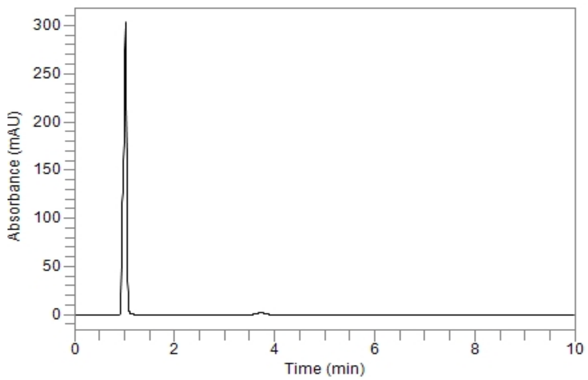

Channel Name **320:10:400:10**

Component Name	Avg RT (min)	RT %RSD	Avg Area	Area %RSD	Avg Height	Height %RSD	Avg Final Amount	Final %RSD
	N/A	N/A	N/A	N/A	N/A	N/A	N/A	N/A

UA std 5 ug/mL : 282:10:400:10 : 1

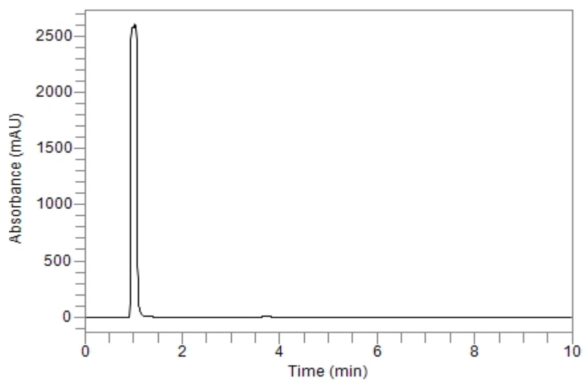

UA std 5 ug/mL : 282:10:400:10 : 2

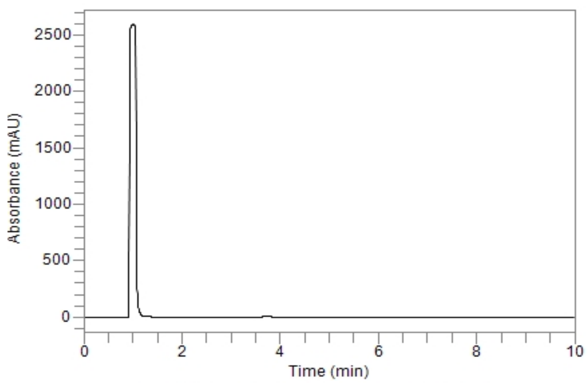

UA std 5 ug/mL : 282:10:400:10 : 3

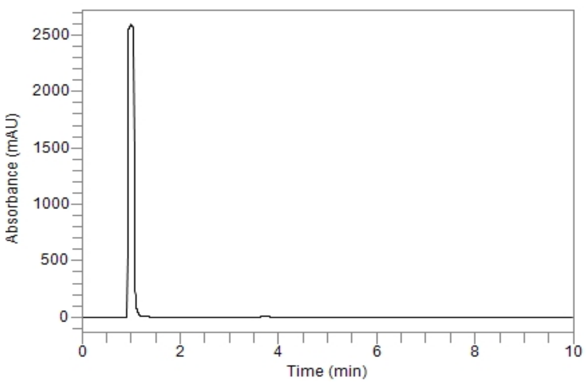

UA std 5 ug/mL : 282:10:400:10 : 4

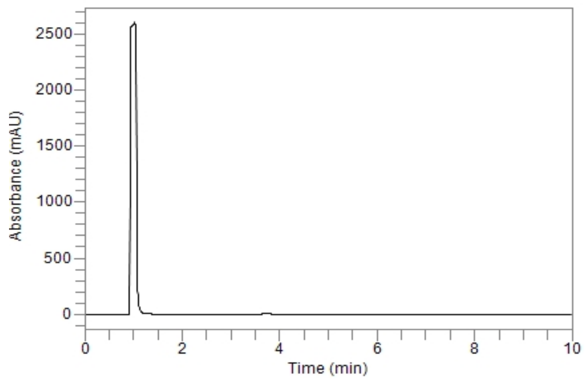

UA std 5 ug/mL : 282:10:400:10 : 5

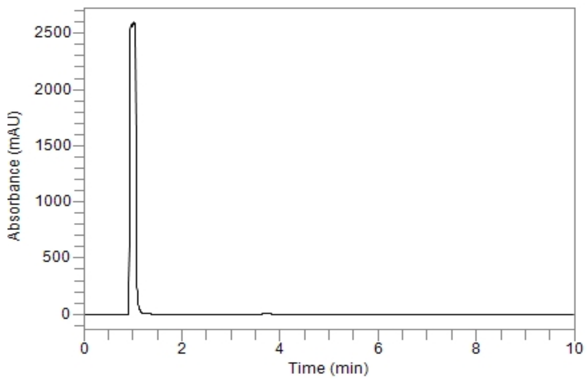

Channel Name **282:10:400:10**

Component Name	Avg RT (min)	RT %RSD	Avg Area	Area %RSD	Avg Height	Height %RSD	Avg Final Amount	Final %RSD
Usnic Acid	3.730	0.129	73,474.0	1.448	7,214.1	1.677	5.9175	1.235

Copy 11-10-

Sample Replicate Report - Multi-Channel

Sample Name	UA std 10 ug/mL
Batch Group/Name	UMF Ovidius/20211109 Usnea barbata oil extract - Copy 11-10-2021 08-56-41 - Copy 11-10-2021 09-15-52 - [illegible]

Acquisition Method	20211103 Usnic Acid Oil		
Processing Method	20211103 Usnic Acid Oil		
Instrument Name	HPLC-PDA Plus		
Vial Number	6	Operator	dan.rambu
Acquisition Date	11/9/2021	Chromera Version	4.2.0.6415

UA std 10 ug/mL : 320:10:400:10 : 1

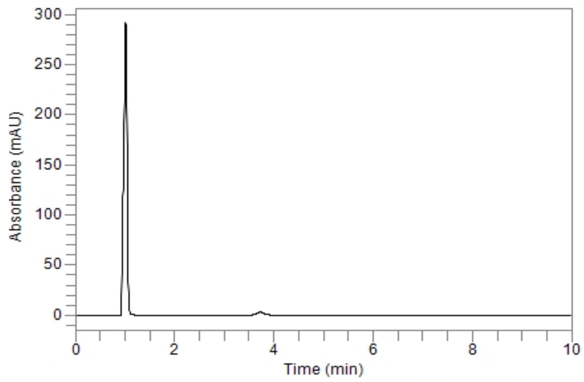

UA std 10 ug/mL : 320:10:400:10 : 2

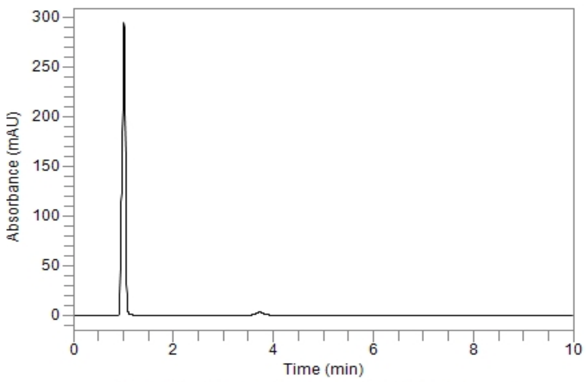

UA std 10 ug/mL : 320:10:400:10 : 3

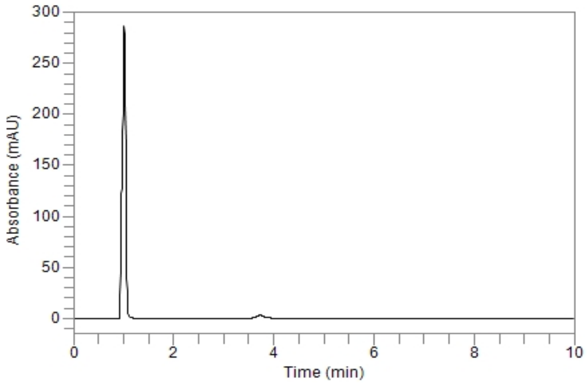

UA std 10 ug/mL : 320:10:400:10 : 4

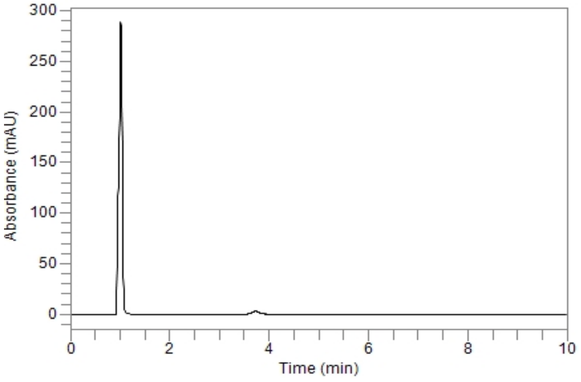

UA std 10 ug/mL : 320:10:400:10 : 5

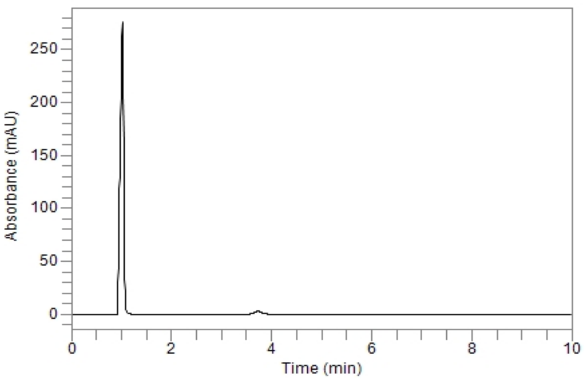

Channel Name **320:10:400:10**

Component Name	Avg RT (min)	RT %RSD	Avg Area	Area %RSD	Avg Height	Height %RSD	Avg Final Amount	Final %RSD
	N/A	N/A	N/A	N/A	N/A	N/A	N/A	N/A

UA std 10 ug/mL : 282:10:400:10 : 1

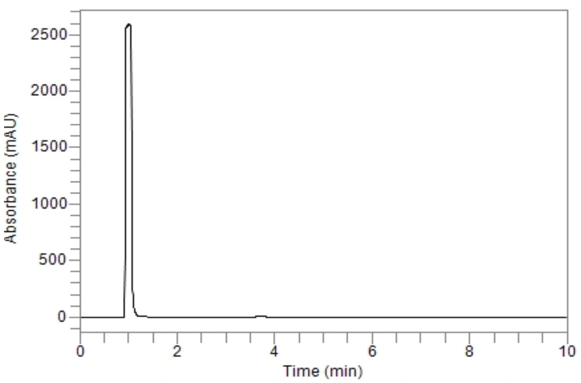

UA std 10 ug/mL : 282:10:400:10 : 2

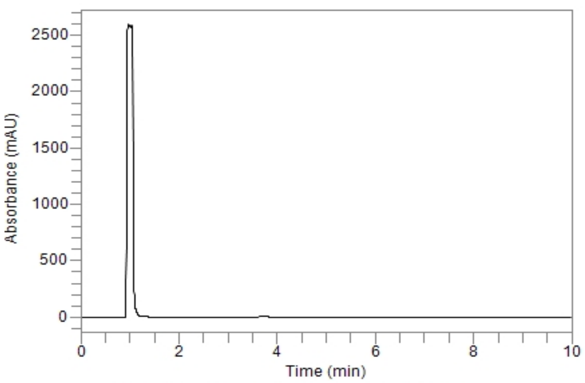

UA std 10 ug/mL : 282:10:400:10 : 3

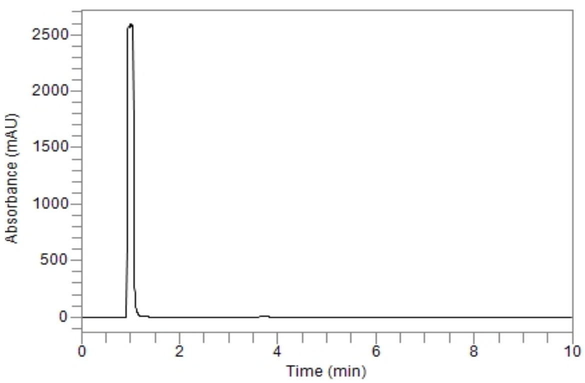

UA std 10 ug/mL : 282:10:400:10 : 4

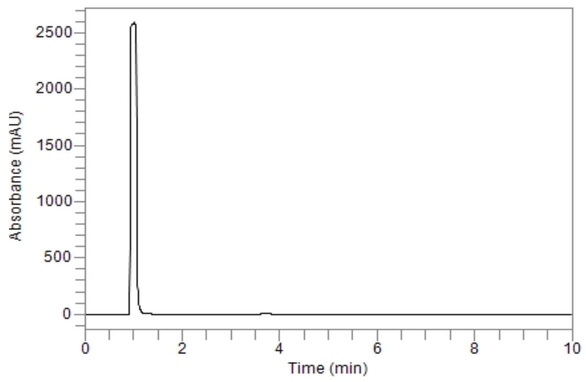

UA std 10 ug/mL : 282:10:400:10 : 5

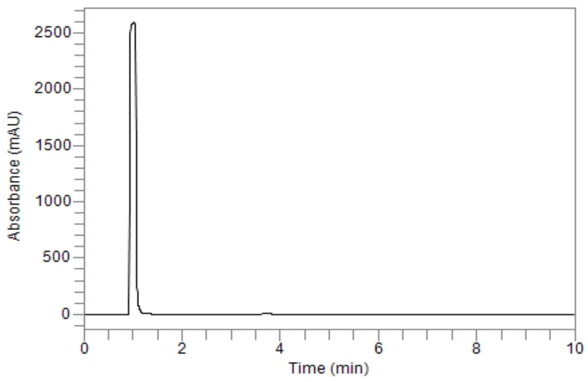

Channel Name **282:10:400:10**

Component Name	Avg RT (min)	RT %RSD	Avg Area	Area %RSD	Avg Height	Height %RSD	Avg Final Amount	Final %RSD
Usnic Acid	3.730	0.046	101,020.2	1.206	9,864.4	0.554	7.8107	1.072

Copy 11-10-

Sample Replicate Report - Multi-Channel

Sample Name	UA std 25 ug/mL
Batch Group/Name	UMF Ovidius/20211109 Usnea barbata oil extract - Copy 11-10-2021 08-56-41 - Copy 11-10-2021 09-15-52 - (

Acquisition Method	20211103 Usnic Acid Oil		
Processing Method	20211103 Usnic Acid Oil		
Instrument Name	HPLC-PDA Plus		
Vial Number	7	Operator	dan.rambu
Acquisition Date	11/9/2021	Chromera Version	4.2.0.6415

UA std 25 ug/mL : 320:10:400:10 : 1

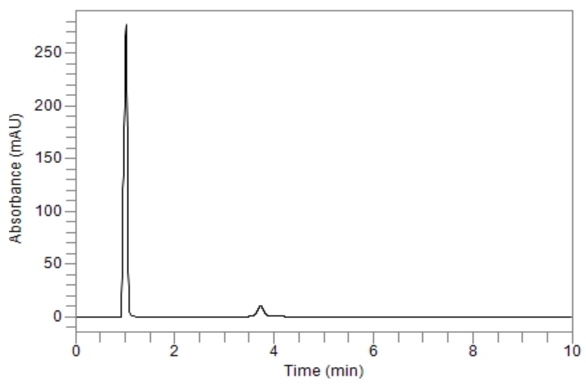

UA std 25 ug/mL : 320:10:400:10 : 2

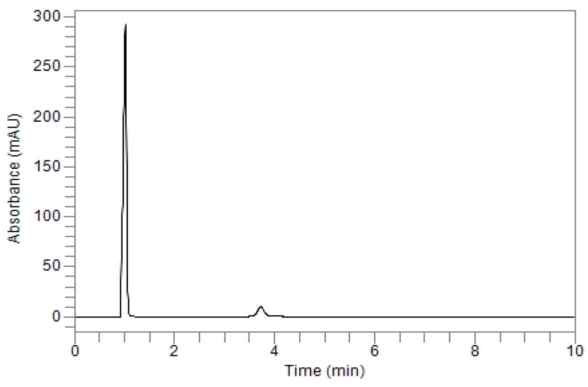

UA std 25 ug/mL : 320:10:400:10 : 3

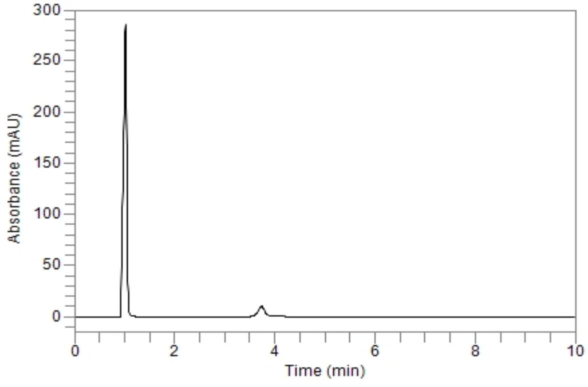

UA std 25 ug/mL : 320:10:400:10 : 4

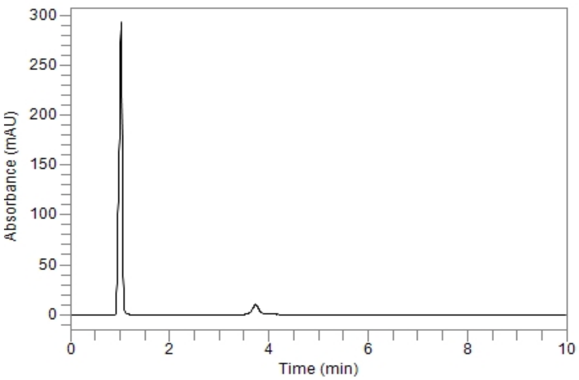

UA std 25 ug/mL : 320:10:400:10 : 5

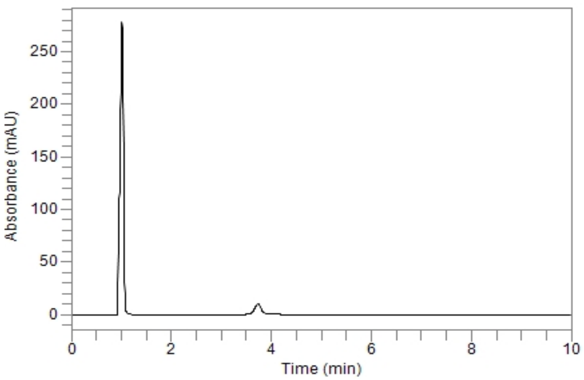

Channel Name **320:10:400:10**

Component Name	Avg RT (min)	RT %RSD	Avg Area	Area %RSD	Avg Height	Height %RSD	Avg Final Amount	Final %RSD
	N/A	N/A	N/A	N/A	N/A	N/A	N/A	N/A

UA std 25 ug/mL : 282:10:400:10 : 1

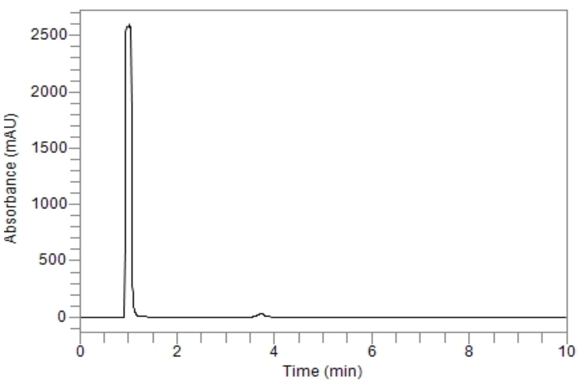

UA std 25 ug/mL : 282:10:400:10 : 2

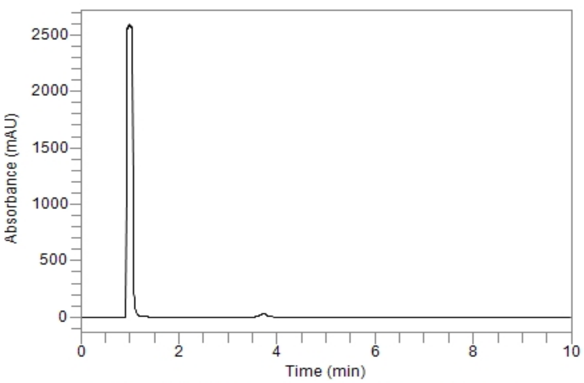

UA std 25 ug/mL : 282:10:400:10 : 3

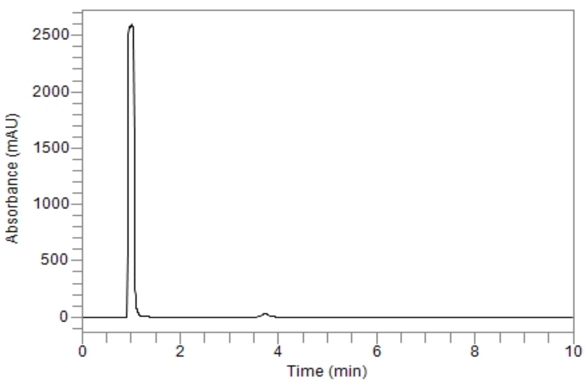

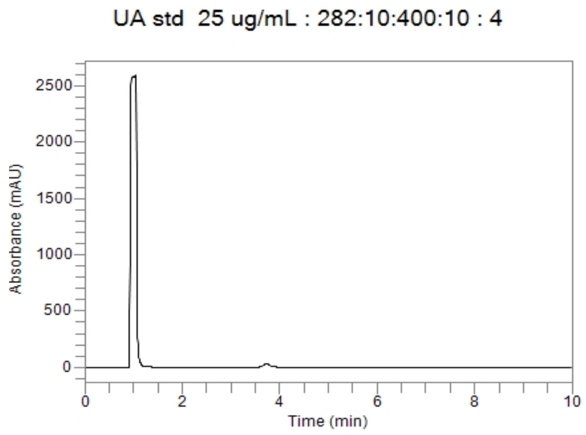

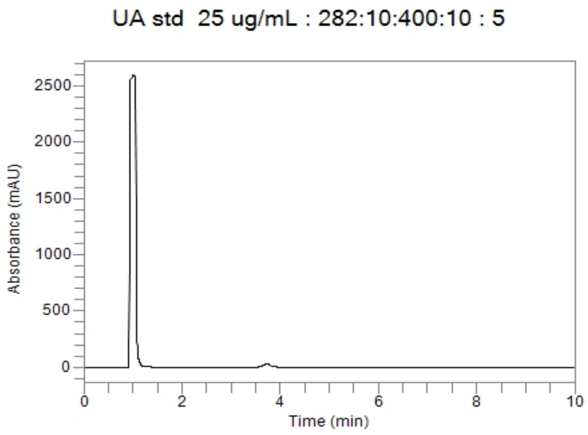

Channel Name **282:10:400:10**

Component Name	Avg RT (min)	RT %RSD	Avg Area	Area %RSD	Avg Height	Height %RSD	Avg Final Amount	Final %RSD
Usnic Acid	3.732	0.087	342,590.6	1.985	32,796.4	0.574	24.4142	1.915

Copy 11-10-

Sample Replicate Report - Multi-Channel

Sample Name	UA std 50 ug/mL
Batch Group/Name	UMF Ovidius/20211109 Usnea barbata oil extract - Copy 11-10-2021 08-56-41 - Copy 11-10-2021 09-15-52 - (

Acquisition Method	20211103 Usnic Acid Oil		
Processing Method	20211103 Usnic Acid Oil		
Instrument Name	HPLC-PDA Plus		
Vial Number	8	Operator	dan.rambu
Acquisition Date	11/9/2021	Chromera Version	4.2.0.6415

UA std 50 ug/mL : 320:10:400:10 : 1

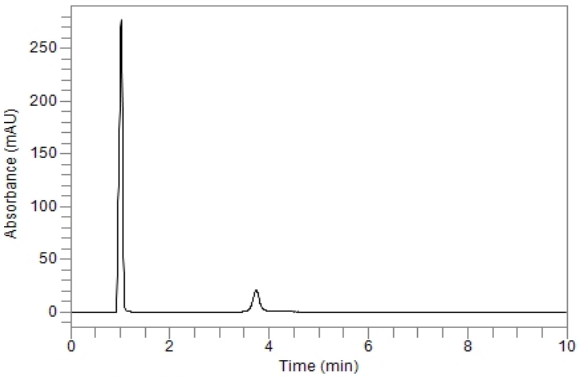

UA std 50 ug/mL : 320:10:400:10 : 2

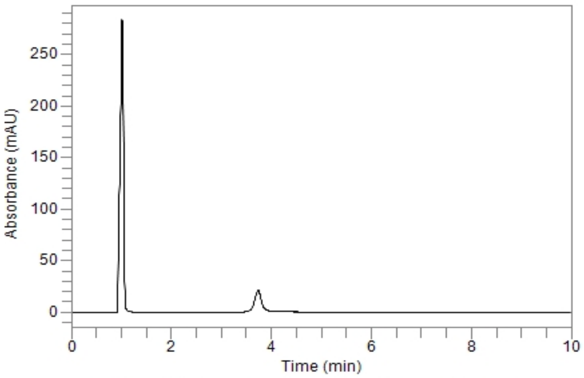

UA std 50 ug/mL : 320:10:400:10 : 3

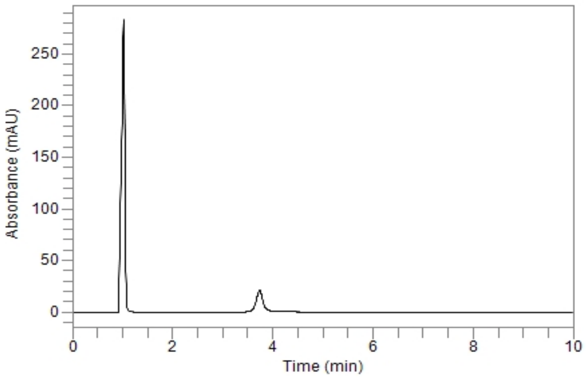

UA std 50 ug/mL : 320:10:400:10 : 4

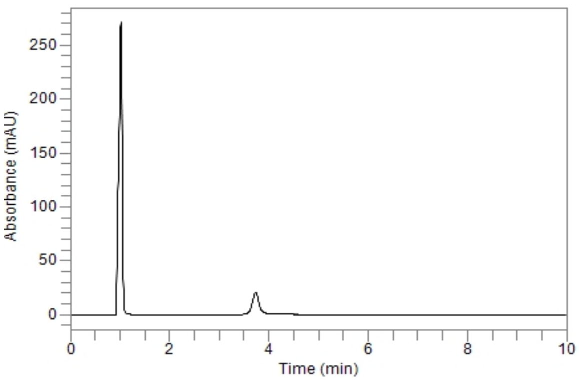

UA std 50 ug/mL : 320:10:400:10 : 5

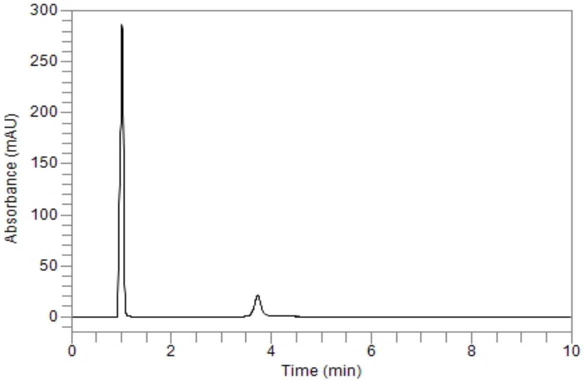

Channel Name **320:10:400:10**

Component Name	Avg RT (min)	RT %RSD	Avg Area	Area %RSD	Avg Height	Height %RSD	Avg Final Amount	Final %RSD
	N/A	N/A	N/A	N/A	N/A	N/A	N/A	N/A

UA std 50 ug/mL : 282:10:400:10 : 1

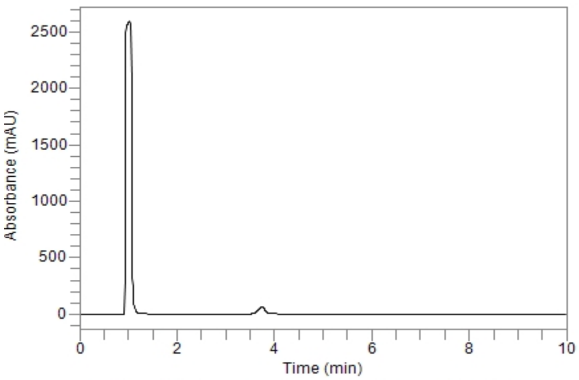

UA std 50 ug/mL : 282:10:400:10 : 2

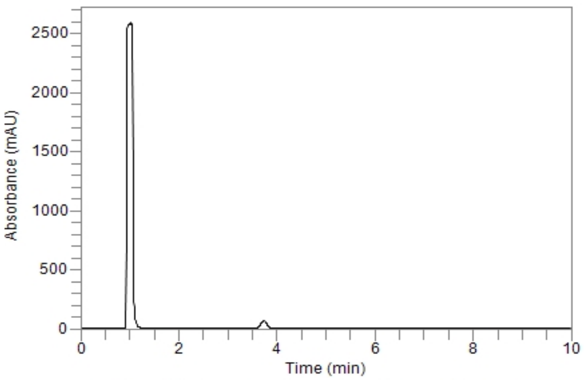

UA std 50 ug/mL : 282:10:400:10 : 3

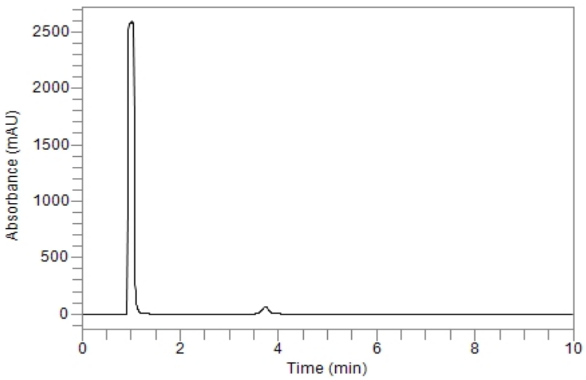

UA std 50 ug/mL : 282:10:400:10 : 4

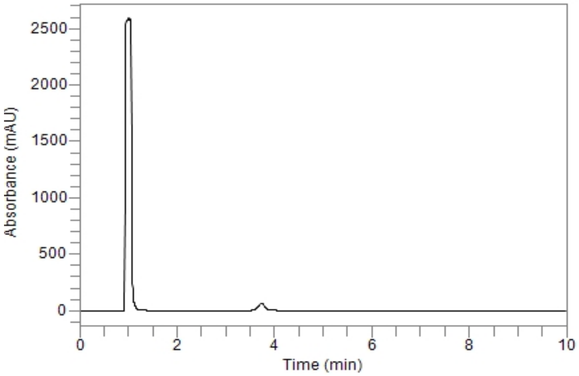

UA std 50 ug/mL : 282:10:400:10 : 5

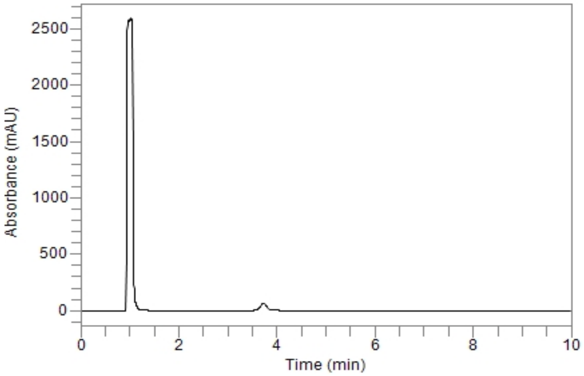

Channel Name **282:10:400:10**

Component Name	Avg RT (min)	RT %RSD	Avg Area	Area %RSD	Avg Height	Height %RSD	Avg Final Amount	Final %RSD
Usnic Acid	3.735	0.115	712,154.0	2.152	67,653.2	0.845	49.8148	2.115

Copy 11-10-

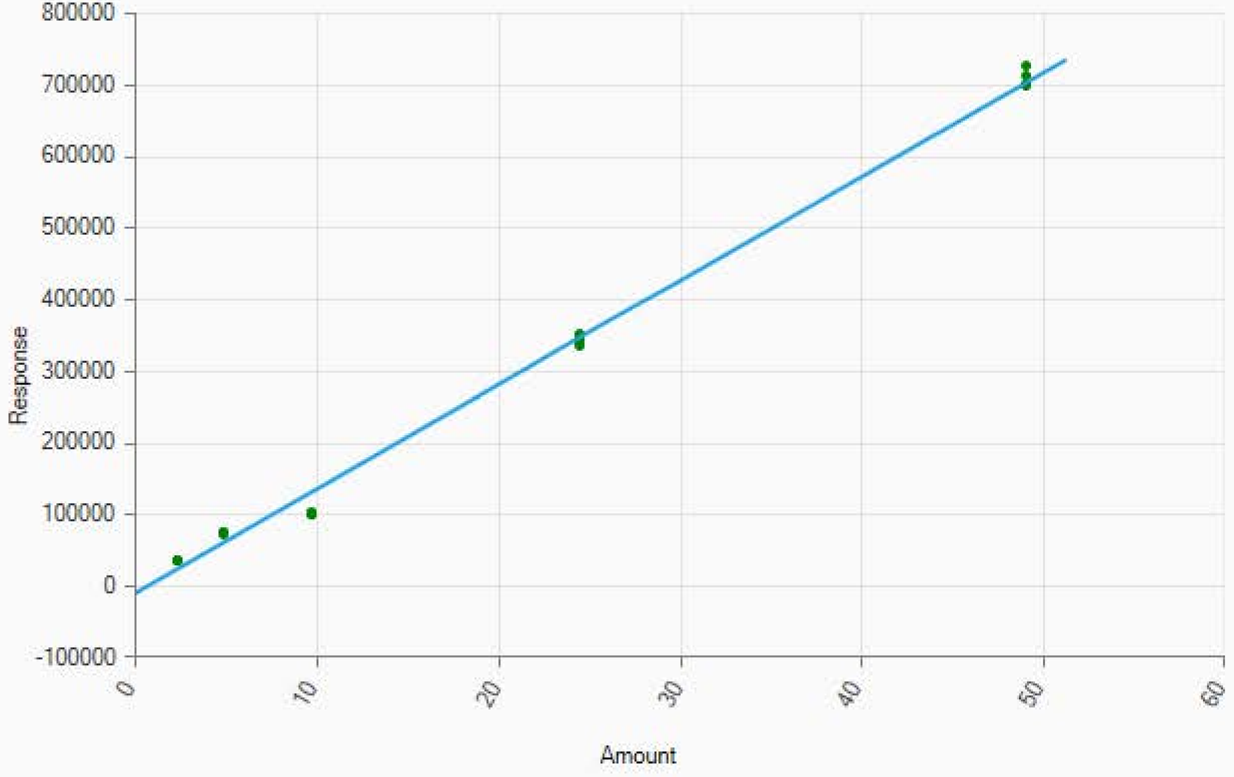

Component Definition

Name:	282:10:400:10: Usnic Acid
Calibration Type:	Linear - Ignore Origin
Scaling:	None
Weighting:	None
Internal Standard:	

Calibration Curve

Equation: y = 14.54939E+3 x + -12.62135E+3

Curve Statistics:

r^2 = 0.99507

%RSE = 22.6
