# Supplementary material for: Antioxidant, Cytotoxic, and Rheological Properties of Canola Oil Extract of Usnea barbata (L.) Weber ex F.H. Wigg from Călimani Mountains, Romania
Source: Plants (Basel). 2022 Mar 23;11(7):854. doi: 10.3390/plants11070854 (PMC9002375; doi:10.3390/plants11070854)
Supplement: Supplementary file 1 [file plants-11-00854-s001.zip › Figure S1..pdf]

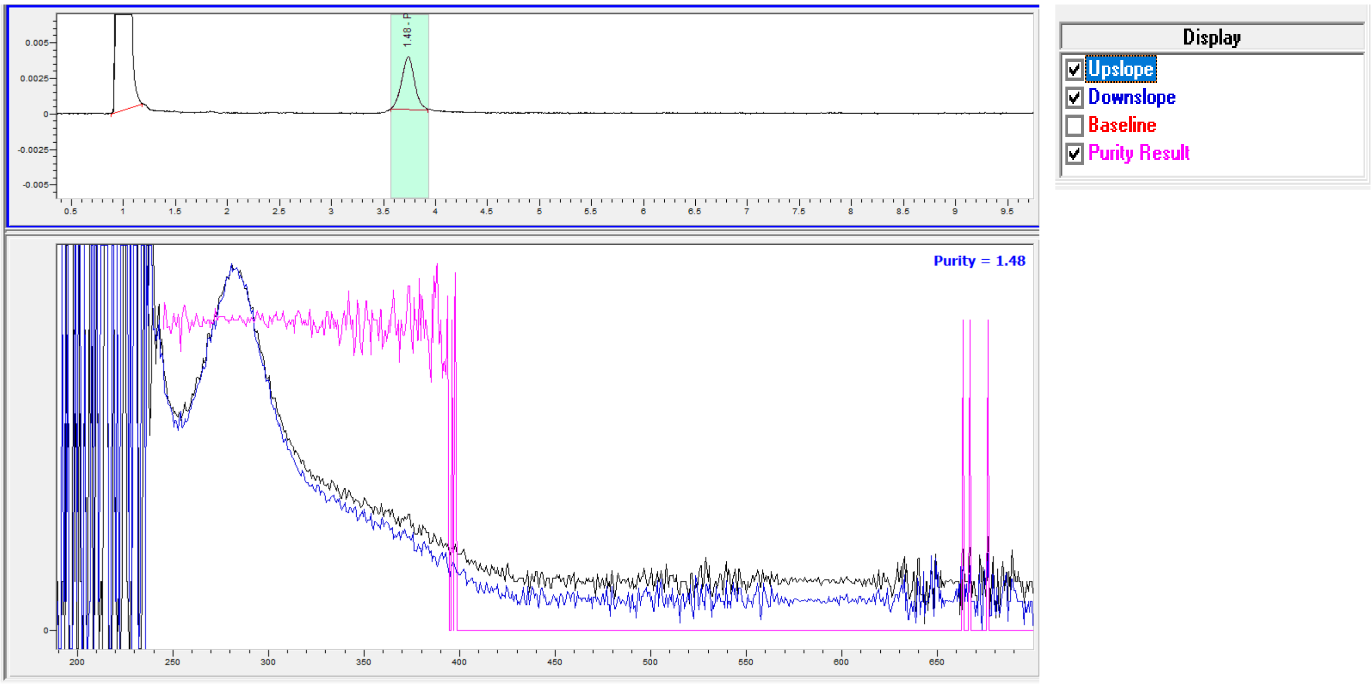

Figure S1. Spectral reprocessing on 240–700 nm range at 15% of peak height for peak identity confirmation and peak purity determination for usnic acid
