# Supplementary figures and images for: Antioxidant, Cytotoxic, and Rheological Properties of Canola Oil Extract of Usnea barbata (L.) Weber ex F.H. Wigg from Călimani Mountains, Romania
Source: Plants (Basel). 2022 Mar 23;11(7):854. doi: 10.3390/plants11070854 (PMC9002375; doi:10.3390/plants11070854)

Acetone : 282:10:400:10 : 1

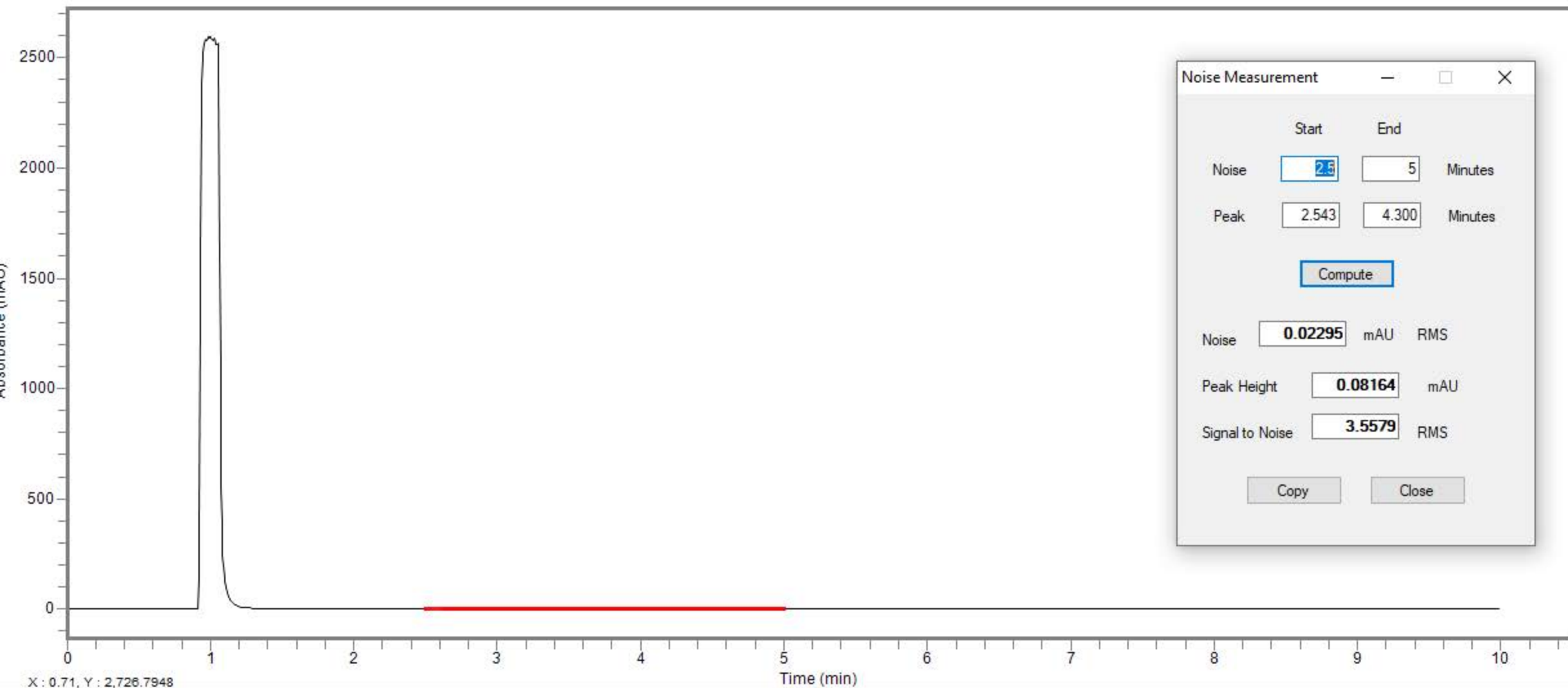

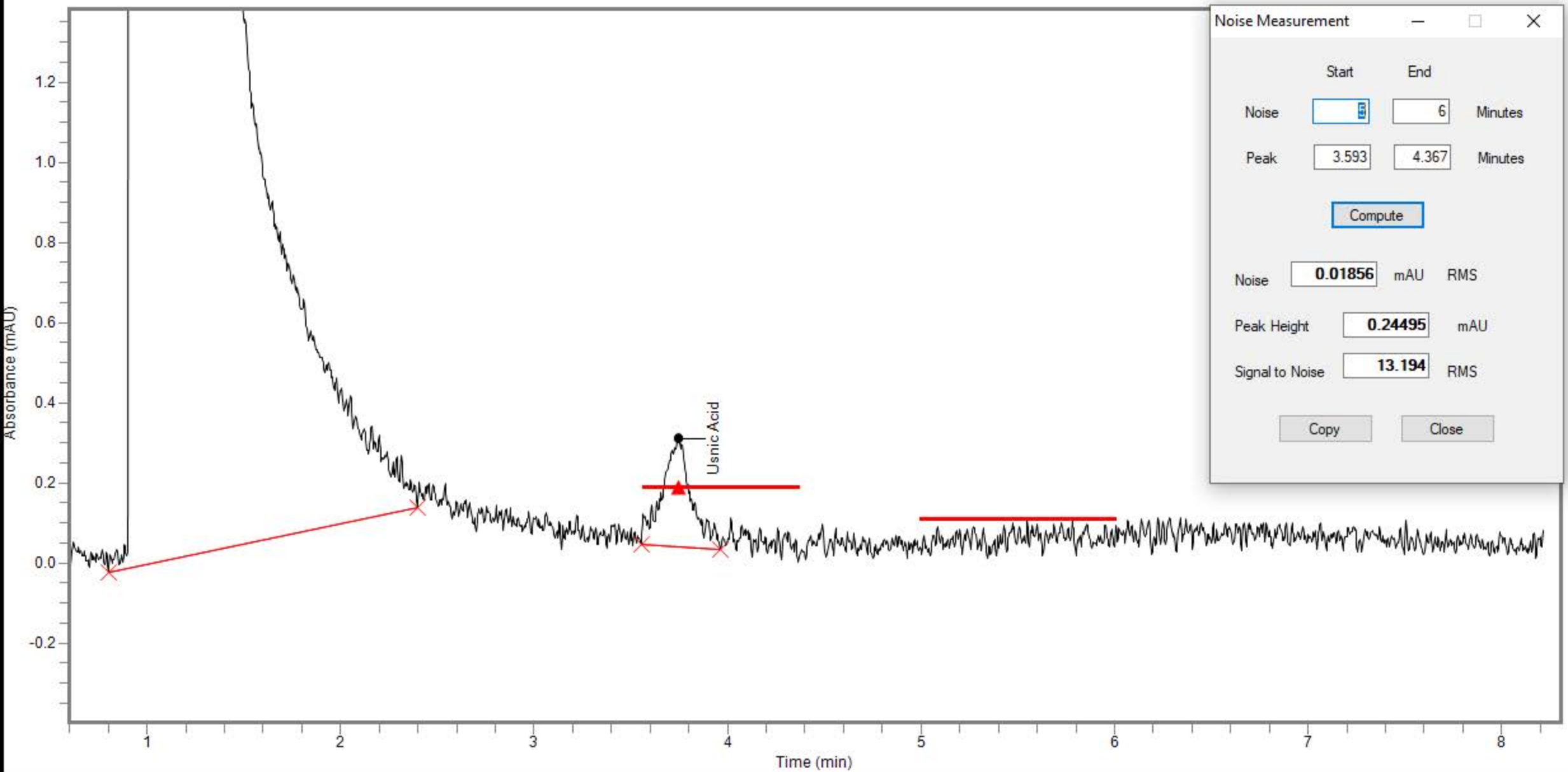

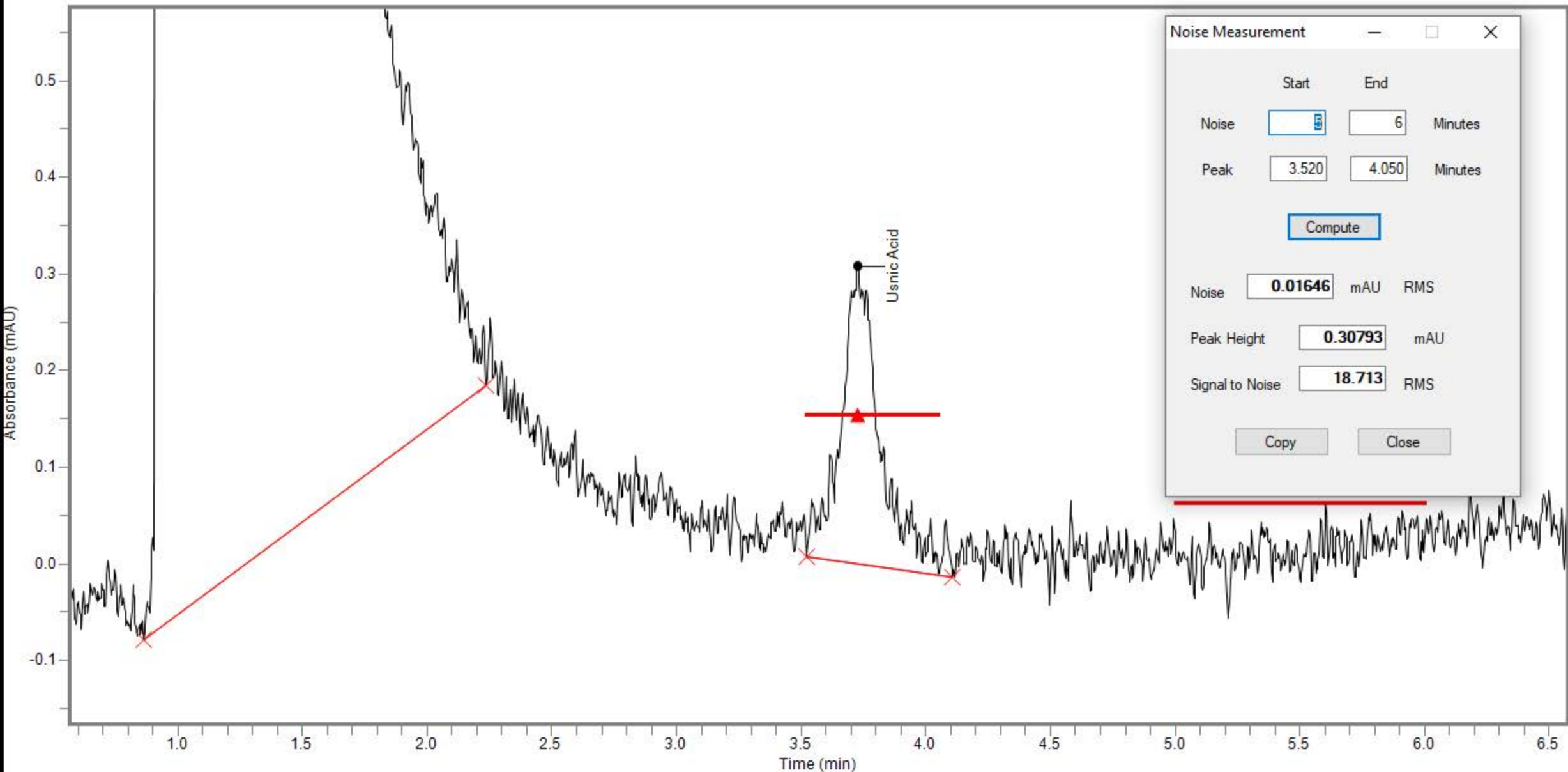

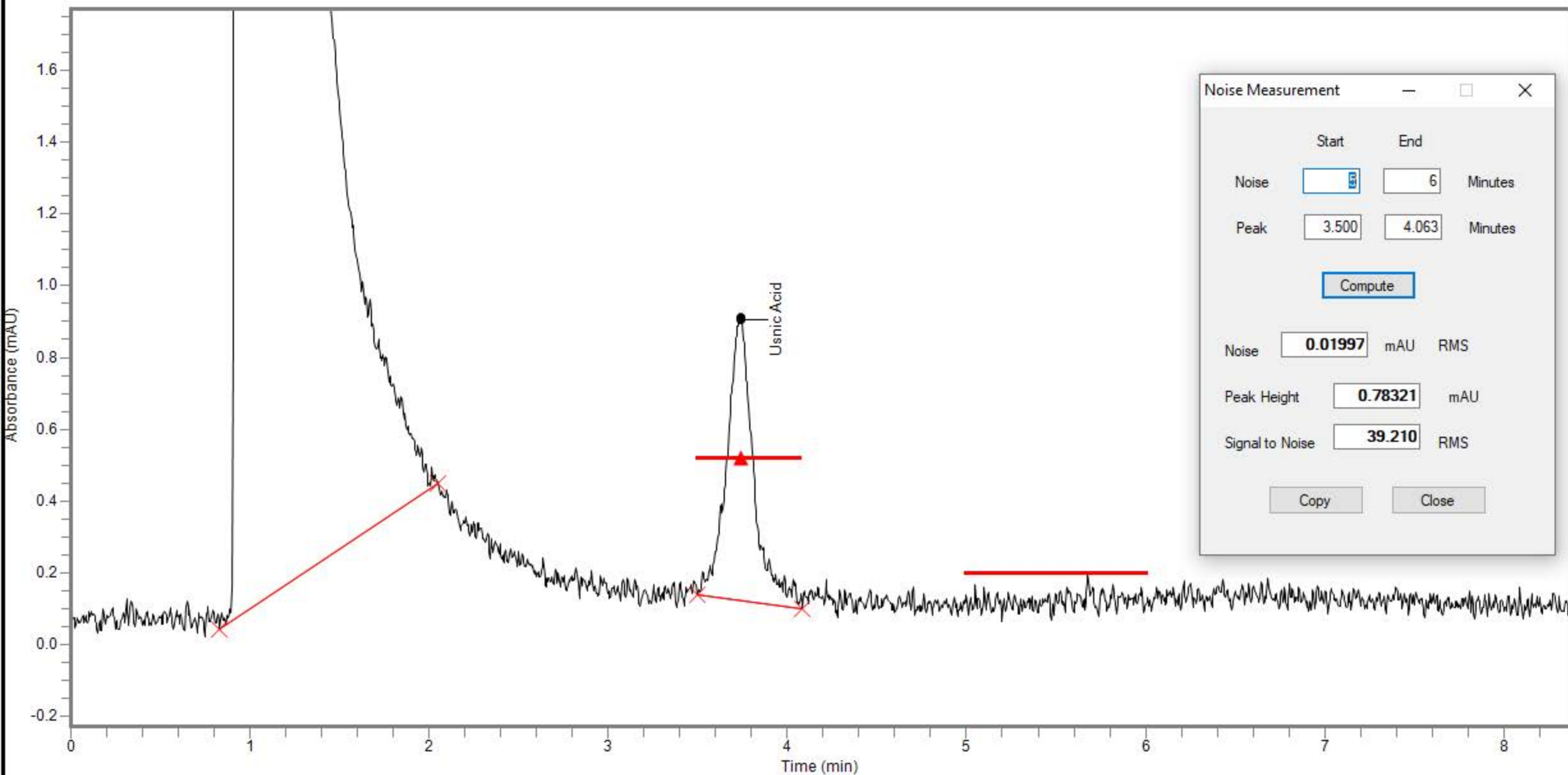

Supplement: Supplementary file 1 [file plants-11-00854-s001.zip › UHPLC LOD, LOQ.pdf]

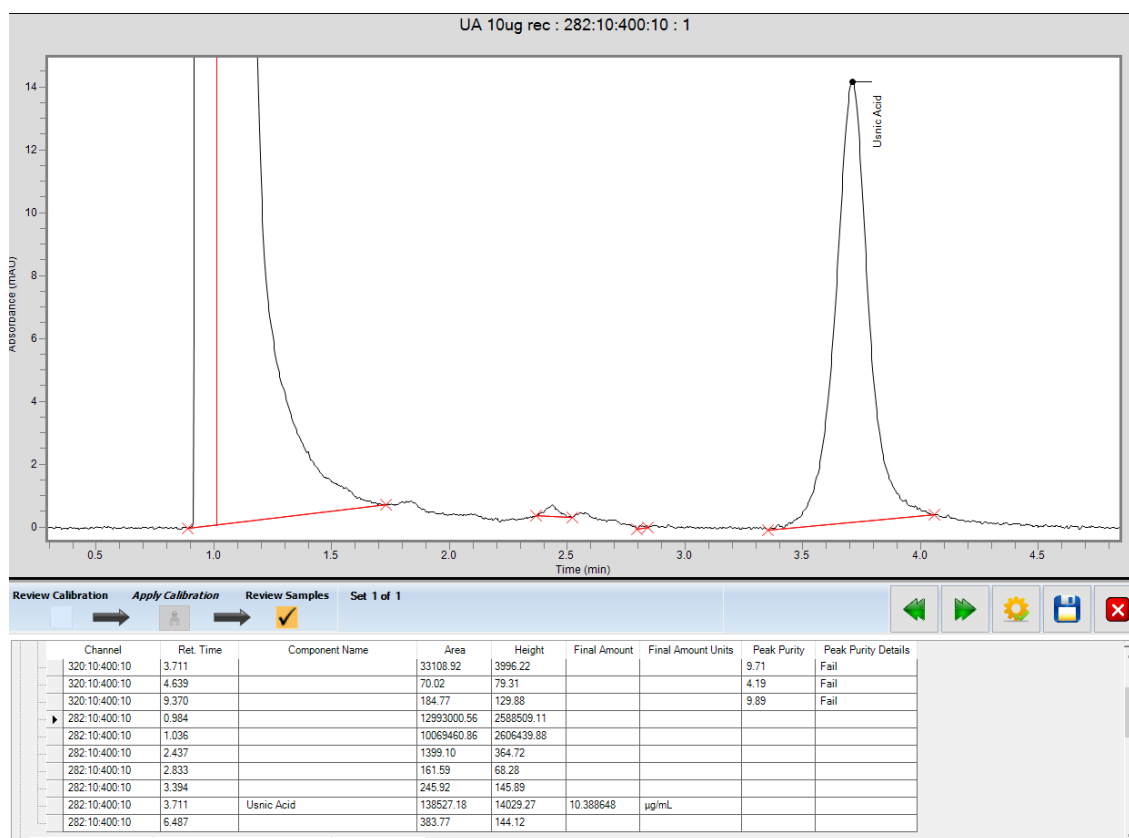

(a)

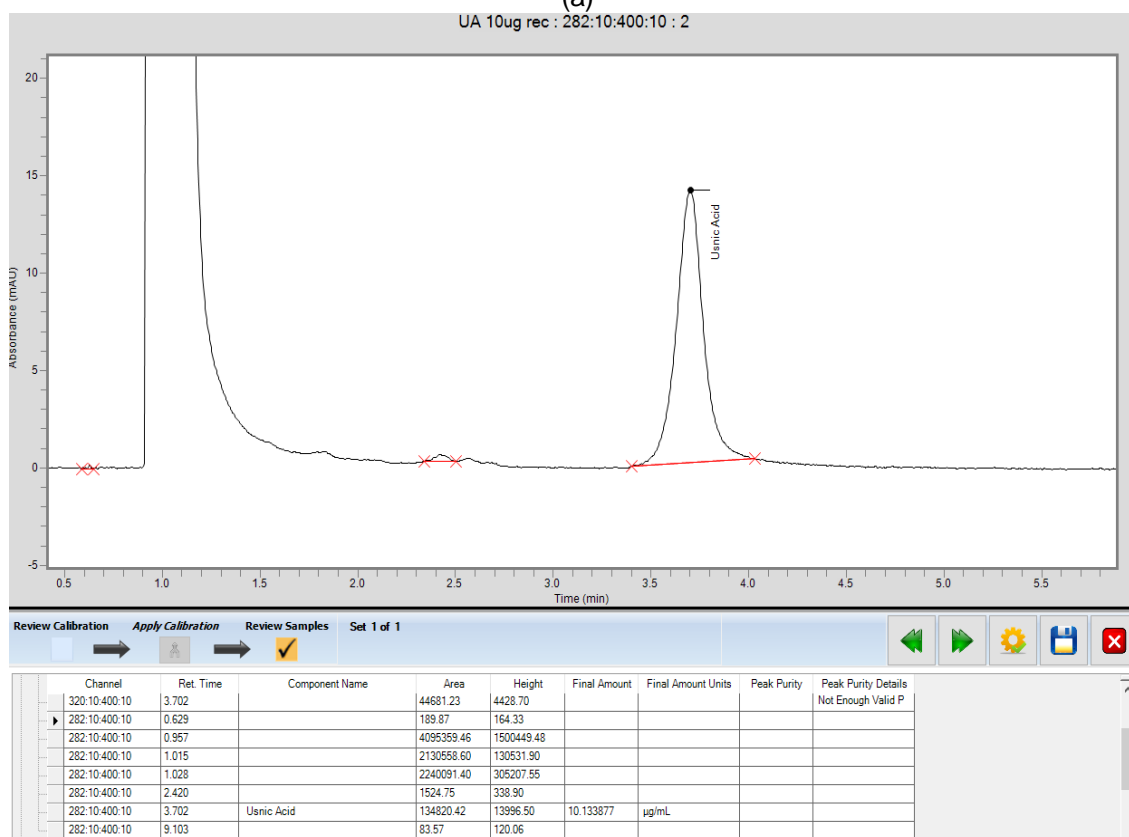

(b)

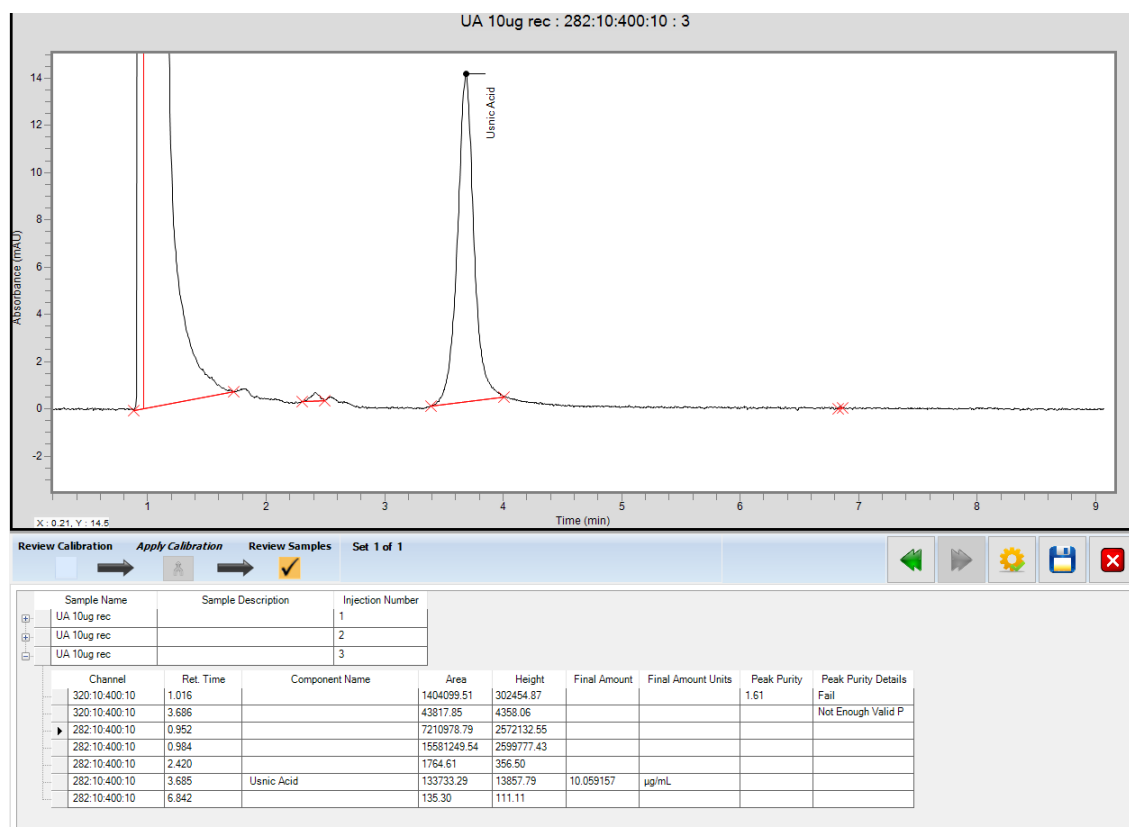

(c)

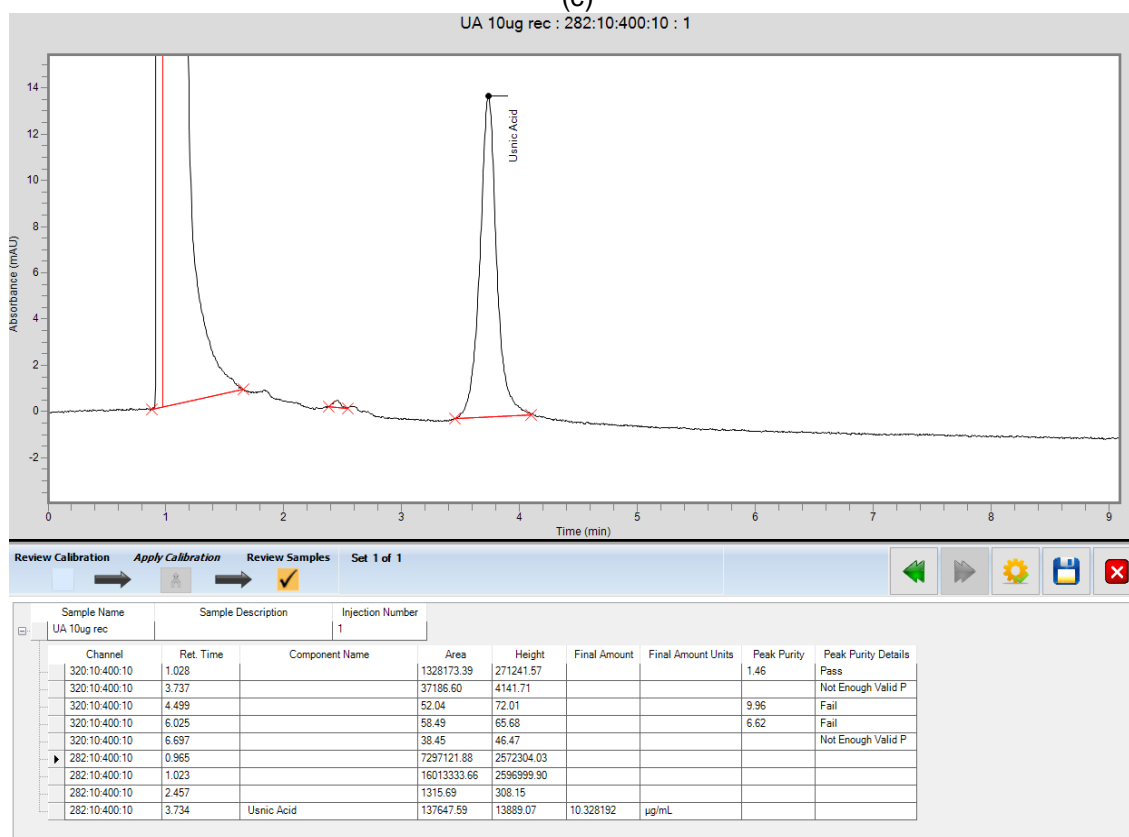

(d)

Figure S2. UA 10 µg recovery (a) Spike solution 1; (b) Spike solution 2; (c) Spike solution 3; (d) Spike solution 4.

Supplement: Supplementary file 1 [file plants-11-00854-s001.zip › Figure S2..pdf]

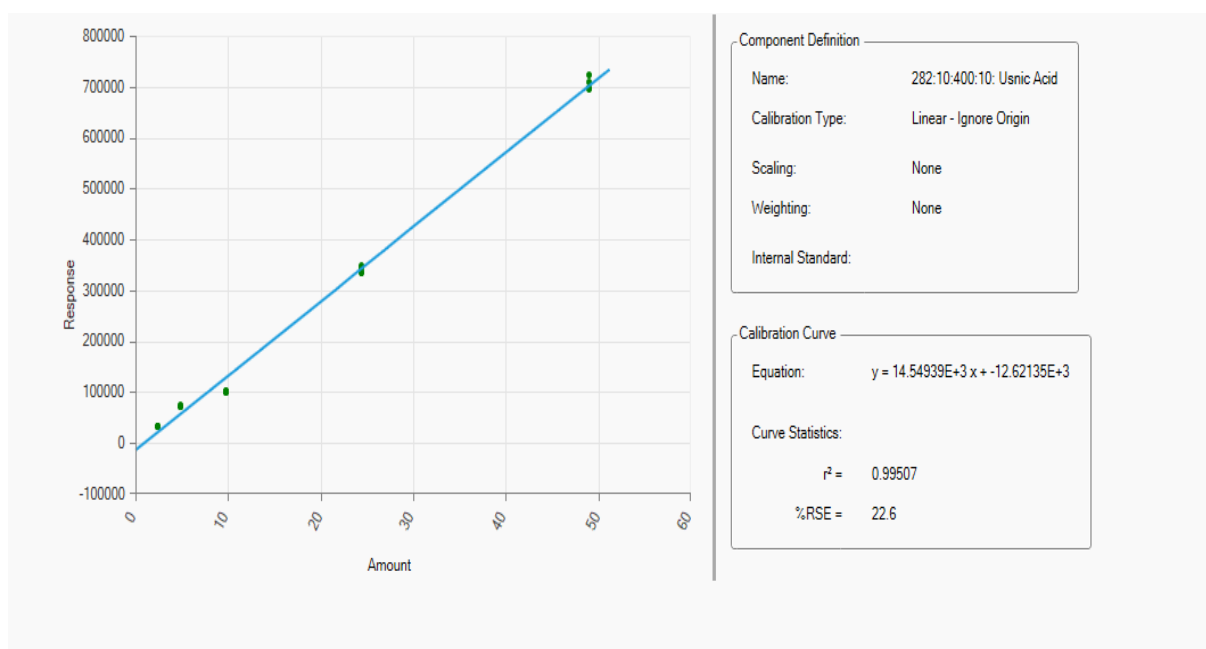

**Figure S3.** The calibration curve of usnic acid standard

Supplement: Supplementary file 1 [file plants-11-00854-s001.zip › Figure S3..pdf]

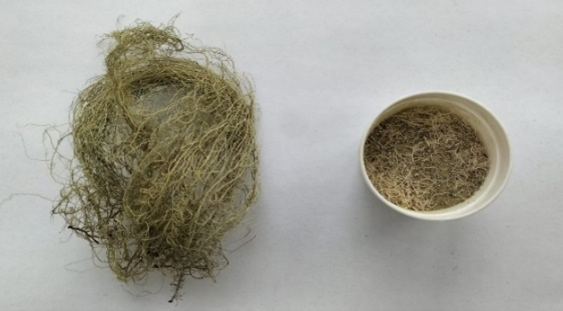

Supplement: Supplementary file 1 [file plants-11-00854-s001.zip › Figure S4, a,b.png]

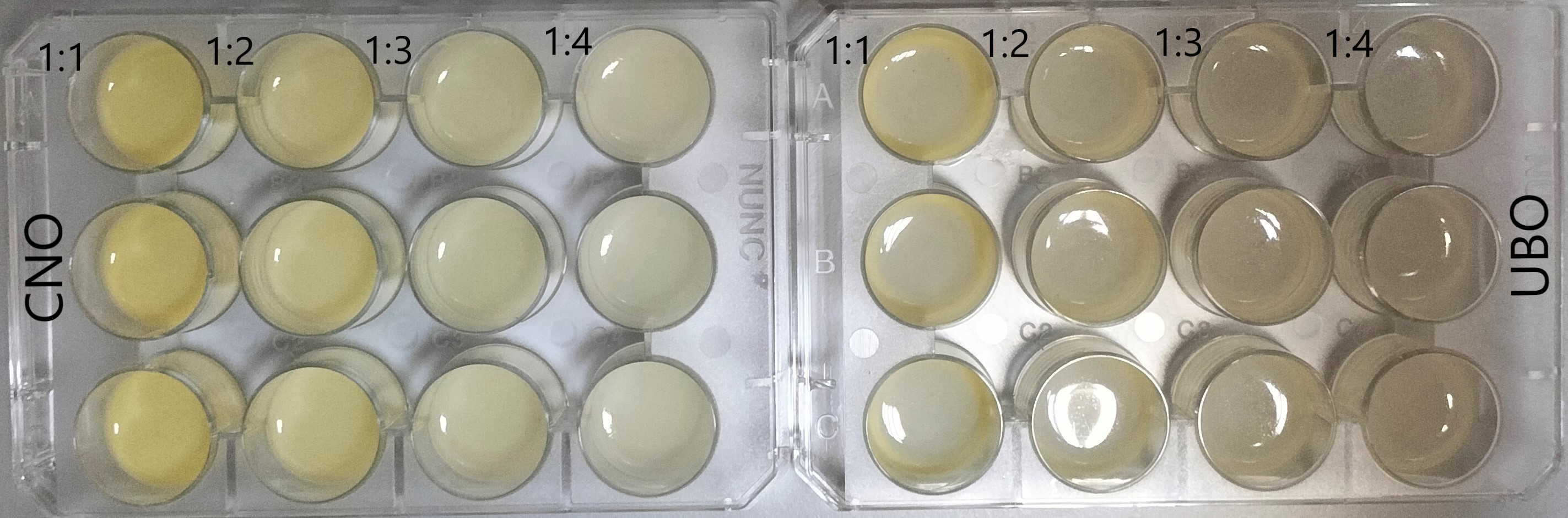

Supplement: Supplementary file 1 [file plants-11-00854-s001.zip › Figure S5..jpg]
